# DNA and RNA interference mechanisms by CRISPR-Cas surveillance complexes

**DOI:** 10.1093/femsre/fuv019

**Published:** 2015-04-30

**Authors:** André Plagens, Hagen Richter, Emmanuelle Charpentier, Lennart Randau

**Affiliations:** 1 Max Planck Institute for Terrestrial Microbiology, Karl-von-Frisch Strasse 10, 35043 Marburg, Germany; 2 Helmholtz Centre for Infection Research, Department of Regulation in Infection Biology, Braunschweig 38124, Germany; 3 The Laboratory for Molecular Infection Medicine Sweden (MIMS), Umeå Centre for Microbial Research (UCMR), Department of Molecular Biology, Umeå University, Umeå 90187, Sweden; 4 Hannover Medical School, Hannover 30625, Germany

**Keywords:** viruses, CRISPR, DNA interference, guide crRNAs, Cascade, Cas9, tracrRNA, ribonucleoprotein complexes

## Abstract

The CRISPR (clustered regularly interspaced short palindromic repeats)-Cas (CRISPR-associated) adaptive immune systems use small guide RNAs, the CRISPR RNAs (crRNAs), to mark foreign genetic material, e.g. viral nucleic acids, for degradation. Archaea and bacteria encode a large variety of Cas proteins that bind crRNA molecules and build active ribonucleoprotein surveillance complexes. The evolution of CRISPR-Cas systems has resulted in a diversification of *cas* genes and a classification of the systems into three types and additional subtypes characterized by distinct surveillance and interfering complexes. Recent crystallographic and biochemical advances have revealed detailed insights into the assembly and DNA/RNA targeting mechanisms of the various complexes. Here, we review our knowledge on the molecular mechanism involved in the DNA and RNA interference stages of type I (Cascade: CRISPR-associated complex for antiviral defense), type II (Cas9) and type III (Csm, Cmr) CRISPR-Cas systems. We further highlight recently reported structural and mechanistic themes shared among these systems.

## Abstract (precis)

This review details and compares the assembly and the DNA/RNA targeting mechanisms of the various surveillance complexes of prokaryotic CRISPR-Cas immune systems.

## INTRODUCTION

**Figure 1.**
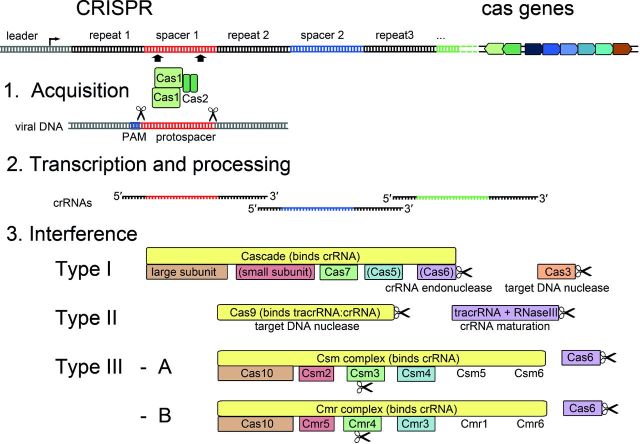
CRISPR-Cas systems and conserved stages of CRISPR-Cas activity. The general organization of a CRISPR-Cas locus is indicated. In the first stage of CRISPR-Cas activity—acquisition—the universal proteins Cas1 and Cas2 recognize viral DNA that is flanked by a PAM. The protospacer is excised and integrated as a spacer sequence into the extending CRISPR array. The CRISPR array is transcribed from the leader sequence and processed into mature crRNAs that are incorporated into crRNP surveillance complexes. The Cas protein composition of the complexes is schematically depicted for the three different CRISPR-Cas types. Nucleases are indicated by scissors and proteins proposed to fulfill similar roles are colored accordingly.

Viruses are the most abundant biological entities on Earth and have been found to quickly adapt to their respective hosts in any environmental niche (Wasik and Turner 2013). As a consequence, all organisms have evolved a wide range of specific antiviral measures that are classified as either innate or adaptive immunity systems. Prokaryotes protect themselves from viruses by using distinct innate immune systems, e.g. restriction-modification systems, the modification of receptors or abortive infection (Samson *et al.*^2013^). In addition, RNA-mediated adaptive immune systems, termed CRISPR-Cas are prevalently distributed in bacterial and archaeal genomes (Barrangou *et al.*^2007^). CRISPR-Cas has largely evolved into three types (type I, II, III) of systems that are characterized by several conserved features and by a common functionality. The hallmarks of these systems are CRISPR loci that consist of a series of short identical repeat sequences separated by spacer sequences that are mostly originated from mobile genetic elements including viruses (Mojica *et al.*^2005^; Pourcel, Salvignol and Vergnaud 2005; Grissa, Vergnaud and Pourcel 2009) (Fig. 1). In most cases, CRISPR arrays are genome encoded, but they can also be located on plasmids or megaplasmids (Mojica *et al.*^2005^; Godde and Bickerton 2006). Additionally, *cas* genes are often located in close proximity to the CRISPR loci and the encoded Cas proteins fulfill essential roles in three defined stages of CRISPR-Cas-mediated immunization and the protection of the prokaryotic cell (Jansen *et al.*^2002^; Haft *et al.*^2005^; Makarova *et al.*2011b). In the first stage of immunity, termed acquisition, a protospacer sequence of DNA from a defective viral infection is recognized by a complex of the universal Cas proteins Cas1–Cas2 and inserted into the host CRISPR array, generating a new spacer as well as a duplication of the repeat in the extended locus (Barrangou *et al.*^2007^; Swarts *et al.*^2012^; Yosef, Goren and Qimron 2012; Savitskaya *et al.*^2013^; Nunez *et al.*^2014^, ^2015^). Protospacer selection in type I and type II relies on short conserved sequences (2–5 bp) that are defined as the protospacer adjacent motif (PAM). PAM sequences are not required for type III systems. Thus, any sequence that flanks correct PAMs has the potential to be integrated into the CRISPR array on the host genome (Mojica *et al.*^2009^; Shah *et al.*^2013^). In most cases, CRISPR immunity is activated by the transcription of the repeat-spacer array into a long precursor-crRNA (pre-crRNA) that is further processed into short crRNA molecules (Carte *et al.*^2008^; Hale *et al.*^2008^; Haurwitz *et al.*^2010^). The mature crRNAs that contain the acquired viral sequence are then incorporated into CRISPR ribonucleoprotein complexes (crRNP) and guide the sequence-specific degradation of viral DNA or RNA upon a second infection (Brouns *et al.*^2008^; Sapranauskas *et al.*^2011^; Rouillon *et al.*^2013^). In this review, we focus mostly on recent structural and mechanistic insights into the crRNP complexes that promote DNA or RNA interference. The diversification of core Cas components and the recruitment of specific Cas proteins to the complexes have resulted in significant differences in structure and composition of these crRNP assemblies in bacteria and archaea. Yet, several mechanistic principles are uniformly shared.

## CRISPR-CAS COMPLEXITY

In the last decade, the identification and classification of *cas* genes illustrates the evolution of diversified CRISPR-Cas systems. A first link between CRISPR loci and the accompanying *cas* genes, *cas1*-*cas4*, was uncovered in 2002 (Jansen *et al.*^2002^). Comparative analyses of available microbial genomes detected numerous associated *cas* genes and revealed major differences in their sequence and organization (Makarova *et al.*^2002^, ^2006^; Haft *et al.*^2005^). Comprehensive analyses of *cas* sequences in bacteria and archaea demonstrated their highly dynamic evolution. Recombined *cas* gene sequences were identified, indicating a frequent horizontal transfer of single genes within a CRISPR-Cas module or even entire *cas* gene cassettes (Millen *et al.*^2012^; Takeuchi *et al.*^2012^; Jiang *et al.*^2013^). Such directed evolution has resulted in a rather challenging classification as well as functional comparison, and was recently highlighted by the branching of Cas proteins into more than 100 families (Koonin and Makarova 2013). Computational classification studies combined with sequence information and prediction of structural similarities have led to the identification of major crRNP building blocks and the detection of many shared domains among Cas protein families in the different CRISPR-Cas types (Makarova *et al.*2011a; Koonin and Makarova 2013). These defined building blocks compose the three functional stages of the CRISPR-Cas immunity: (i) spacer insertion, (ii) crRNA processing, (iii) crRNP assembly and target cleavage (Makarova, Wolf and Koonin 2013) (Fig. 1).

## DIVERSIFICATION OF CRISPR-CAS INTERFERENCE MECHANISMS

The current nomenclature classifies the CRISPR-Cas systems of bacteria and archaea into three main types (I, II and III) and 11 subtypes (I-A to F, II-A to C, III-A to B) based on phylogenetic and often limited functional studies (Makarova *et al.*2011b; Chylinski, Le Rhun and Charpentier 2013; Koonin and Makarova 2013). The types are defined by a conserved signature protein (Cas3 in type I, Cas9 in type II and Cas10 in type III) and mainly differ in crRNP assembly and target cleavage mechanisms. All type I crRNP complexes are termed Cascade (CRISPR-associated complex for antiviral defense), while in type II the stand-alone Cas9 nuclease is responsible for target cleavage. Type III systems encode the Csm (III-A) or Cmr (III-B) crRNP complex (Makarova *et al.*2011b) (Fig. 1). Only Cas1 and Cas2, which are involved in spacer acquisition, are conserved in the majority of CRISPR-Cas systems (Yosef, Goren and Qimron 2012; Makarova, Wolf and Koonin 2013). In the following sections, we will focus on the distinctions of crRNA processing, crRNP assembly and target cleavage within the different CRISPR-Cas types.

### Composition of type I Cascade effector complexes

#### The type I-E DNA interference complex

**Figure 2.**
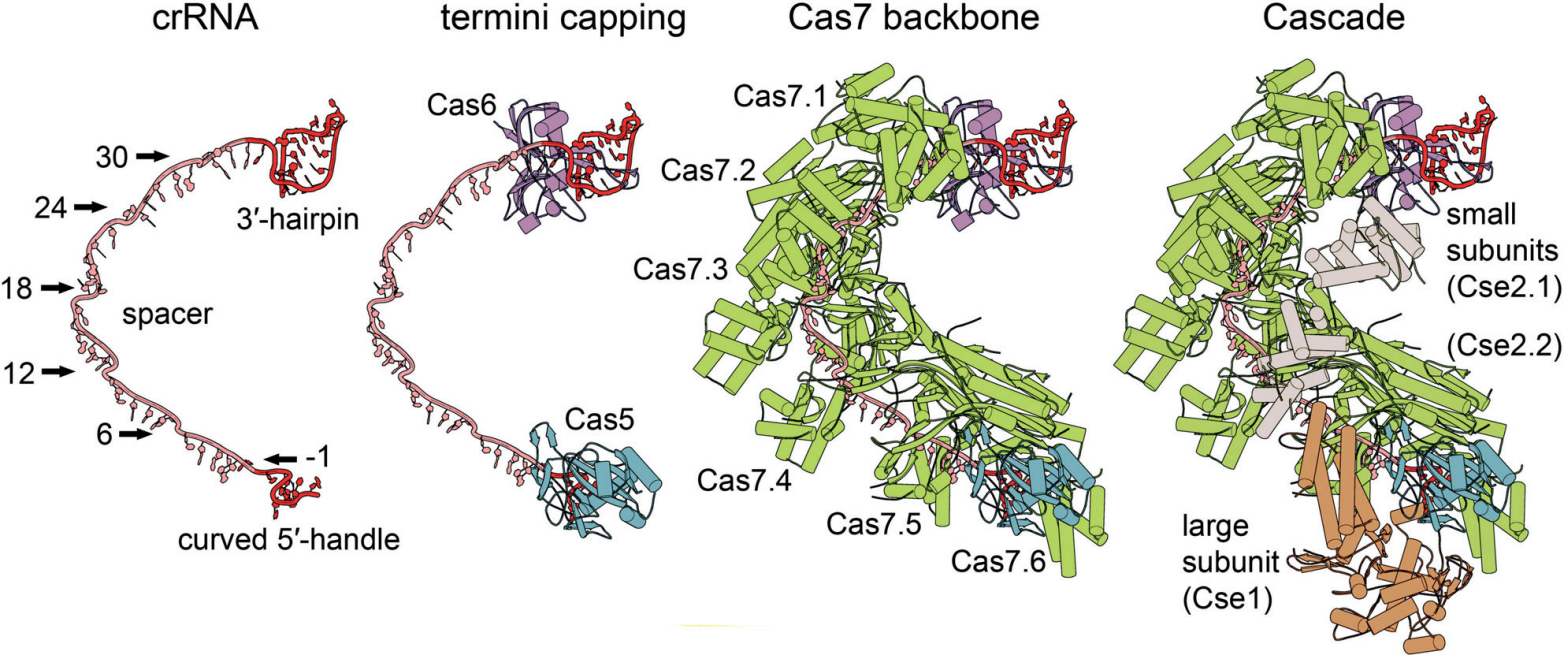
Assembly of the type I-E Cascade structure. The I-E Cascade complex has a seahorse-shaped structure and consists of 11 protein subunits ((Cse1)_1_-(Cse2)_2_-(Cas5)_1_-(Cas7)_6_-(Cas6e)_1_) and a single 61-nt crRNA (pdb: 4TVX). Cas6e is tightly bound to the 3^′^ stem-loop structure of the mature crRNA and positioned at the head of the complex. Cas5e directly caps the 5^′^ handle of the crRNA, which leads to the hook-like structure of the crRNA. The structure of Cas5 and Cas7 displays a conserved palm-thumb domain arrangement, highlighting the intertwined assembly of the Cascade backbone. The thumb of either Cas5 or each of the six Cas7 subunits (Cas7.1-Cas7.6) kinks the crRNA at position −1 in the 5^′^ handle and every sixth position in the spacer sequence, and buries the base between the thumb and the palm of the adjacent Cas7 subunit. The two small Cse2 subunits (Cse2.1–Cse2.2) are connected to the crRNA backbone via protein:protein interactions to the Cas7 subunits. The large subunit Cse1 is positioned at the Cascade tail and interacts with Cas5, Cas7 and Cse2.

The type I-E system is widespread among bacterial phyla, common for Gammaproteobacteria, and rarely found in individual euryarchaeal organisms (Brouns *et al.*^2008^; Makarova, Wolf and Koonin 2013). This is the best studied type I CRISPR-Cas system and was originally found in *Escherichia coli*. This type has been termed I-E (for *Escherichia*) and encodes the first DNA interference complex to be named Cascade (Brouns *et al.*^2008^). The I-E Cascade is built up by a single 61-nt long crRNA and five different Cas proteins in an uneven subunit stoichiometry: (Cse1)_1_-(Cse2)_2_-(Cas5)_1_-(Cas7)_6_-(Cas6e)_1_ resulting in a total crRNP mass of 405 kDa (Brouns *et al.*^2008^; Jore *et al.*^2011^). The mature 61-nt crRNA is generated via specific cleavage by the Cas6e endoribonuclease within the repeat sequence of a pre-crRNA transcript (Gesner *et al.*^2011^; Sashital, Jinek and Doudna 2011). A former nomenclature of type I-E refers to Cas6e as Cse3 or CasE. For the sake of clarity, we will use Cas6e and related terminology throughout this review. Cas6e-mediated cleavage of repeat sequences yields a 8-nt 5^′^ handle with a hydroxyl group, a 32-nt spacer sequence and a 21-nt 3^′^ hairpin structure with a cyclic 2^′^-3^′^ phosphate end (Jore *et al.*^2011^). After cleavage, Cas6e stays bound to the 3^′^ hairpin of the mature crRNA (Niewoehner, Jinek and Doudna 2014). Cascade then assembles with Cas5 (known as Cas5e or CasD) binding to the 5^′^ handle of the crRNA and six copies of Cas7 (known as Cse4 or CasC) binding to the spacer sequence (Brouns *et al.*^2008^; Jore *et al.*^2011^) (Fig. 2). Two additional proteins are Cse1 (known as CasA) and the Cse2 dimer (known as CasB), which are defined as the large and small Cascade subunits (Brouns *et al.*^2008^). Both subunits are involved in DNA binding, while the large subunits also functions in the target selection (Jore *et al.*^2011^; Wiedenheft *et al.*2011a; Sashital, Wiedenheft and Doudna 2012). In the last step of the interference mechanism, the Cas3 helicase-endonuclease is recruited to degrade the target DNA (Brouns *et al.*^2008^; Westra *et al.*2012b). In *Streptococcus thermophilus*, a rarer orthologous type I-E CRISPR-Cas system was identified. Its structural study revealed a similar crRNP assembly with an observed overrepresentation of the backbone forming Cas7 subunit (Horvath and Barrangou 2010; Sinkunas *et al.*^2011^, ^2013^). The isolated mature crRNA of 61-nt length of this system included a 7-nt 5^′^ handle with a hydroxyl group, a 33-nt spacer sequence and a 21-nt 3^′^ handle with a P_i_ terminal group (Sinkunas *et al.*^2013^).

#### The type I-A DNA interference complex

The type I-A systems are predominantly found in thermophilic archaeal species and are rarely seen in bacteria (Makarova *et al.*2011b). The mechanism of crRNA generation shows clear differences to Cas6e-mediated cleavage, which results in a tight association of crRNA product and Cas6e (Niewoehner, Jinek and Doudna 2014). In contrast, a Cas6 enzyme of type I-A was shown to catalyze a multiple turnover reaction with the release of free crRNA (Sokolowski, Graham and White 2014). Structural studies revealed a dimeric composition of Cas6 with the unstructured repeat RNA substrate bound in a sequence-dependent manner that is essential for catalytic activity (Wang *et al.*^2012^; Reeks *et al.*2013b). The crRNAs have a broad distribution of ∼60–70 nt lengths and contain the characteristic 8 nt 5^′^ handle and a 16–17 nt 3^′^ handle. However, spacer length is more variable ranging from 38 to 44 nts in *Sulfolobus solfataricus* or 37 to 57 nts in *Thermoproteus tenax* (Lintner *et al.*^2011^; Plagens *et al.*^2012^). The 3^′^ handles are often nearly absent in the cellular crRNA pool (Plagens *et al.*^2014^). One explanation for this phenomenon is the observed weak association of Cas6 with the Cascade crRNP, which suggests that Cas6 is not an integral part of the complex. This weak association renders the 3^′^ handle accessible for chemical and/or enzymatic trimming in I-A Cascade that is assembled around the mature crRNA (Plagens *et al.*^2014^; Sokolowski, Graham and White 2014). The core backbone of I-A Cascade is presumably assembled via a multimerization of Cas7 units that form a helical structure along the crRNA and interact with Cas5 (Lintner *et al.*^2011^; Plagens *et al.*^2014^). It has been proposed that the roles of large and small Cascade subunits of the I-A complex are fulfilled by the proteins Cas8a and Csa5, respectively (Lintner *et al.*^2011^; Plagens *et al.*^2014^). The small subunit Csa5 of *The. tenax* was shown to preferably bind to ssDNA, suggesting its involvement in target DNA interaction (Daume, Plagens and Randau 2014). Another characteristic feature of type I-A systems is the split of the signature protein Cas3 into two proteins containing either the helicase (Cas3^′^) or the nuclease domain (Cas3^′^^′^). In contrast to I-E Cas3, that is recruited to cleave target DNA, both Cas3^′^ and Cas3^′^^′^ subunits are an integral part of the I-A Cascade crRNP (Plagens *et al.*^2014^).

#### The type I-B DNA interference complex

The type I-B systems are present in diverse archaeal and bacterial lineages and show some characteristics of both type I-A and type I-C systems (Makarova *et al.*2011b). Mature crRNAs of bacterial and archaeal species are generated by the endoribonuclease Cas6b and contain a clearly defined 8-nt 5^′^ handle, a 36–40 nt spacer and a 3^′^ handle that is gradually shortened to a minimal 2-nt tag (Richter *et al.*2012b; Elmore *et al.*^2013^; Li *et al.*^2013^; Richter *et al.*^2013^). Type I-B systems further contain Cas7 and Cas5 for building the Cascade backbone which interacts with Cas6b (Brendel *et al.*^2014^). The large subunit is represented by the subtype-specific protein Cas8b and is predicted to have the small subunit fused to its C-terminal end (Makarova *et al.*2011a). In addition, type I-B systems contain Cas3 versions that either have the typical nuclease-helicase fusion arrangement or are occasionally split into two discrete subunits (Makarova *et al.*2011b).

#### The type I-C DNA interference complex

The type I-C system can be found in various bacterial species, predominantly within the Firmicutes group (Haft *et al.*^2005^). This system was characterized in *Bacillus halodurans* and, in contrast to other type I and III systems, does not encode a Cas6 protein for crRNA maturation. Instead, the pre-crRNA cleavage activity is performed by a Cas5 (known also as Cas5d) homolog (Makarova *et al.*2011b). Cas5d was shown to cleave pre-crRNA resulting in a mature crRNA with an 11-nt 5^′^ handle that has a hydroxyl group, a 33-nt spacer sequence and a 21-nt 3^′^ handle containing a cyclic 2^′^-3^′^ phosphate end (Garside *et al.*^2012^; Nam *et al.*2012a). Following the processing event, one Cas5d subunit remains associated with the 3^′^ hairpin handle of the mature crRNA, a mechanistic feature that was also observed for Cas6e (Nam *et al.*2012a). Furthermore, the type I-C Cas5 is proposed to act as a bifunctional protein and a second subunit that presumably binds to the 5^′^ handle of the crRNA, similar to Cas5 from *E. coli* type I-E (Nam *et al.*2012a). The I-C Cascade crRNP also contains the backbone-forming subunit Cas7 (known as Csd2) and the large subunit Cas8 (known as Cas8c or Csd1) (Nam *et al.*2012a). Computational analyses predicted a fusion of large and small subunits in the type I-C Cas8 protein with its C-terminal region showing homology to the small subunit Cse2 of type I-E (Makarova *et al.*2011a; Punetha, Sivathanu and Anand 2014). The overall architecture of the I-C Cascade resembles I-E Cascade with a mass of ∼400 kDa and a proposed stoichiometry (Cas8)_1_-(Cas7)_6_-(Cas5)_2_ (Nam *et al.*2012a). The type I-C system additionally encodes Cas3 with a conserved nuclease-helicase domain involved in the final target DNA degradation step (Makarova *et al.*2011b).

#### The type I-D DNA interference complex

Type I-D systems are mainly found in cyanobacteria and euryarchaeal species (Makarova, Wolf and Koonin 2013). They feature the type I signature protein Cas3, but also a Cas10 protein, the signature protein for type III systems, suggesting an evolutionary link between I-C and III-B systems (Makarova *et al.*2011b). The maturation of pre-crRNA in the cyanobacterium *Synechocystis* sp. PCC6803 is mediated by a conserved Cas6 enzyme generating the common 8-nt 5^′^ handle with varying spacer length of 31–47 nt (Scholz *et al.*^2013^). Similar to type I-A and I-B, crRNAs of type I-D show a stepwise trimming of the 3^′^ end that might indicate a release of Cas6 from the crRNA after pre-crRNA cleavage (Hein *et al.*^2013^). The *Thermofilum pendens* I-D Cas7 protein was crystallized, revealing structural similarities with other Cas7 proteins and ssRNA binding activity. These features suggest that it builds up the I-D crRNP backbone (Makarova *et al.*2011a; Hrle *et al.*^2014^). A second protein involved in backbone formation might be the subtype-specific protein Csc1 that was previously grouped into the Cas5 family (Makarova *et al.*2011a). The large subunit of I-D Cascade is predicted to be the protein Cas10d, which shows a similar structural organization as the large subunits of the Cas8 family and Cse1 of type I-E (Makarova *et al.*2011a). Additionally, the HD nuclease domain of Cas3 (Cas3^′^^′^), essential for target DNA degradation, is fused to Cas10d and the helicase domain (Cas3^′^) is encoded by a stand-alone gene (Makarova *et al.*2011b).

#### The type I-F DNA interference complex

The occurrence of type I-F CRISPR-Cas systems is restricted to bacterial organisms. They are often found in Gammaproteobacteria and show remarkable similarities with the type I-E system (Makarova, Wolf and Koonin 2013). The maturation of crRNAs is mediated by the repeat-specific endoribonuclease Cas6f (known as Csy4) (Haurwitz *et al.*^2010^; Przybilski *et al.*^2011^). The 60-nt mature crRNA is characterized by the typical 8-nt 5^′^ handle, a repeat stem containing a 20-nt 3^′^ handle and 5^′^ OH as well as cyclic 2^′^-3^′^ phosphate termini (Haurwitz *et al.*^2010^; Sternberg, Haurwitz and Doudna 2012). It was observed that Cas6f binds to the 3^′^ handle of the crRNA, which is essential for RNA protection and Cascade I-F crRNP assembly (Haurwitz, Sternberg and Doudna 2012; Sternberg, Haurwitz and Doudna 2012). The I-F Cascade consists of four Cas proteins with a subunit stoichiometry that is similar to other Cascade complexes ((Csy1)_1_-(Cas5)_1_-(Cas7)_6_-(Cas6f)_1_) and a mass of 350 kDa (Wiedenheft *et al.*2011b; Richter *et al.*2012a). The crescent-shaped crRNA backbone is formed by six copies of a Cas7 family protein (known as Csy3), the terminal Cas5 (known as Csy2) at the 5^′^ end and Cas6f at the 3^′^ end of the crRNA (Wiedenheft *et al.*2011b). Sequence analysis and secondary structure predictions could not identify a clear homolog to other large Cascade subunits of the Cas8 family or Cse1, but distinct interactions of Cas5 and Csy1 suggest that Csy1 is fulfilling the role of the large and small subunits for target recognition and DNA binding in the I-F Cascade (Makarova *et al.*2011a; Richter *et al.*2012a). The conserved protein Cas3 shows the typical helicase-nuclease arrangement and is N-terminally fused to a Cas2-like domain (Richter *et al.*2012a). This Cas2-Cas3 fusion forms a complex with Cas1 and also interacts with the I-F Cascade subunits, suggesting a dual function in spacer acquisition and target DNA degradation (Richter *et al.*2012a; Richter and Fineran 2013).

### Composition of type II DNA interference complexes

**Figure 3.**
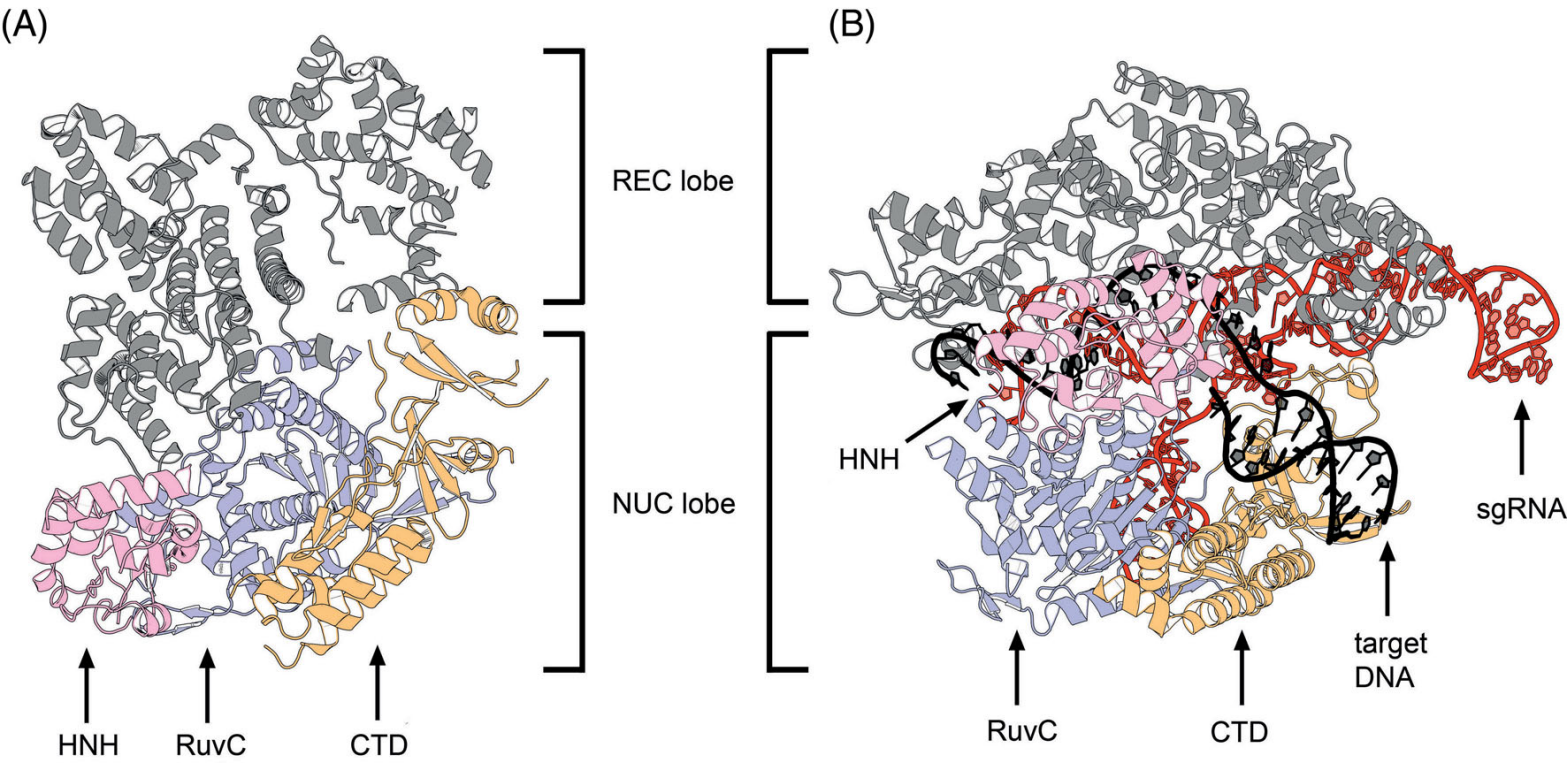
Structures of *S. pyogenes* (Spy) type II-A Cas9. (**A**) Crystal structure of the apoenzyme SpyCas9 resolved at 2.6 Å (pdb: 4CMP) (Jinek *et al.*^2014^). (**B**) Structure of SpyCas9 bound to sgRNA and target DNA resolved at 2.5 Å (pdb: 4UN3) (Anders *et al.*^2014^). While the CTD and the RuvC domains remain in their positions, the REC lobe of Cas9 accommodates its position to facilitate sgRNA and target DNA binding. At the same time, the disordered HNH domain of inactive Cas9 (A) undergoes a conformational change for cleavage of the targeted strand.

While the interference process in types I and III CRISPR-Cas systems involves the formation of a multi-Cas protein complex, Cas9 (formerly Csn1) is the only protein that is required in the DNA targeting event of the type II systems (Barrangou *et al.*^2007^; Garneau *et al.*^2010^; Deltcheva *et al.*^2011^; Sapranauskas *et al.*^2011^). In the first CRISPR-Cas classification, every system possessing a Cas9 protein (formerly COG3515) was grouped into the *Neisseria* (Nmeni) subtype (Haft *et al.*^2005^). The classification currently followed by the community is based on the identification of significant differences among Nmeni-type systems from various organisms, yielding a further sub-classification into subtypes II-A, II-B and II-C (Chylinski, Le Rhun and Charpentier 2013; Koonin and Makarova 2013; Zhang *et al.*^2013^; Chylinski *et al.*^2014^; Fonfara *et al.*^2014^). The discrimination relies mainly on the presence or absence of the adaptation module proteins, Cas4 and Csn2. While Csn2 is found in type II-A, type II-B contains Cas4 and type II-C possesses neither of the two. The type II Cas9 signature proteins show significant diversity in sequence; however, three common features are shared: conserved split HNH and RuvC nuclease domains, an arginine-rich motif and a similar globular architecture (Makarova *et al.*^2006^, 2011b; Chylinski, Le Rhun and Charpentier 2013; Koonin and Makarova 2013; Sampson *et al.*^2013^; Chylinski *et al.*^2014^; Jinek *et al.*^2014^; Nishimasu *et al.*^2014^) (Fig. 3). Based on the predicted nuclease domains, it was proposed that Cas9 would act as a nuclease during the invading nucleic acid interference reaction (Haft *et al.*^2005^; Makarova *et al.*^2006^).

The first interference activity of a type II system was demonstrated in phage challenge experiments. *Streptococcus thermophilus*, containing a type II-A system, acquired immunity against phages following the acquisition of one or more phage sequences that were integrated as spacers in the CRISPR array (Barrangou *et al.*^2007^). It was observed that this acquired bacterial immunity against phage attack was lost upon disruption of the *cas9* gene. Disruption of the *csn2* gene additionally indicated that Cas9 is the only protein required for type II interference (Barrangou *et al.*^2007^; Deveau *et al.*^2008^; Garneau *et al.*^2010^). This was further confirmed when deletions of the conserved adaptation *cas1* and *cas2* genes were shown to also retain the interference function (Sapranauskas *et al.*^2011^; Zhang *et al.*^2013^). *In vivo* experiments later identified dsDNA as the sole target of type II systems (Garneau *et al.*^2010^; Deltcheva *et al.*^2011^; Sapranauskas *et al.*^2011^), in which Cas9 uses HNH and RuvC nuclease domains (Sapranauskas *et al.*^2011^) to cleave the DNA sequence yielding blunt-ended double-strand breaks (Garneau *et al.*^2010^; Magadan *et al.*^2012^).

A second hallmark of the type II systems is the *trans*-activating CRISPR RNA (tracrRNA). While the surveillance machinery of types I and III consists of crRNPs composed of one single crRNA guiding a multi-Cas protein complex to the target nucleic acids, type II systems use a duplex of RNAs (dual-tracrRNA-crRNA) to guide Cas9 to the invading target DNA. The tracrRNA was identified as an abundant small RNA containing an anti-CRISPR repeat and located in the vicinity of type II *cas* genes and CRISPR repeat-spacer arrays (Deltcheva *et al.*^2011^). Initial experiments performed in *S. pyogenes* showed that tracrRNA acts as a *trans*-activator of crRNA maturation (Deltcheva *et al.*^2011^). However, subsequent work demonstrated that tracrRNA also forms a critical component of the Cas9 cleavage complex (Jinek *et al.*^2012^). With respect to maturation, type II systems lack genes encoding Cas6 endoribonucleases that are used by types I and III systems to process crRNAs. Instead, maturation of type II crRNAs involves tracrRNA, Cas9 and the effector endoribonuclease III from the bacterial host. tracrRNA binds via its anti-repeat sequence to each of the repeats of the pre-crRNA forming heteroduplexes that are stabilized by Cas9 (Deltcheva *et al.*^2011^). Each duplex RNA is then recognized and cleaved by the endoribonuclease III, yielding an intermediate form of crRNA and a mature form of tracrRNA (77 nt length) (Deltcheva *et al.*^2011^; Chylinski, Le Rhun and Charpentier 2013; Karvelis *et al.*^2013^). An additional processing of the crRNA, by a so far unknown endo- and/or exonuclease, yields the mature crRNA (44 nt length) composed of spacer sequence in 5^′^ and repeat sequence in 3^′^ (Deltcheva *et al.*^2011^).

With respect to cleavage, it was found that mature crRNA and Cas9 alone were incapable of cleaving target DNA, but the addition of tracrRNA resulted in DNA targeting and cleavage (Jinek *et al.*^2012^). Biochemical analysis of the DNA targeting mechanism of the *S. pyogenes* type II CRISPR-Cas system (Deltcheva *et al.*^2011^) thus demonstrated that Cas9 is a DNA endonuclease that uses a tracrRNA: crRNA duplex to direct DNA cleavage site specifically (Jinek *et al.*^2012^). The HNH domain of Cas9 cleaves the DNA strand that is complementary to the spacer region of crRNA while the RuvC-like domain cleaves the DNA strand opposite the complementary strand (Gasiunas *et al.*^2012^; Jinek *et al.*^2012^).

In a type II-C system (e.g. *Neisseria meningitidis*), an alternative crRNA maturation pathway has been described (Zhang *et al.*^2013^). In this particular system, the repeat units of the CRISPR array contain promoter sequences that can initiate the production of short crRNAs in an endoribonuclease III-independent manner. However, duplex formation of tracrRNA and the short crRNAs were shown to still be required for interference with DNA (Zhang *et al.*^2013^).

A variant Cas9 protein with single-stranded DNA cleavage (nickase) activity can be generated by mutating either the HNH or the RuvC-like domain (Gasiunas *et al.*^2012^; Jinek *et al.*^2012^). Mutating both domains creates an RNA-guided DNA-binding protein with deficient cleavage activity (dead-Cas9) (Gasiunas *et al.*^2012^; Jinek *et al.*^2012^). DNA target recognition requires both base pairing of the crRNA sequence to the protospacer region and the presence of the PAM adjacent to the targeted sequence in the DNA (Gasiunas *et al.*^2012^; Jinek *et al.*^2012^). While it was found that some nucleotide changes in the protospacer sequences can be tolerated, the PAM and the region proximal to the PAM in the protospacer are requirements critical for site-specific Cas9 targeting. Single mutations in the PAM led to escape phages that were not targeted any longer. Changes close to the PAM yielded also non-cleavable targets, indicating the presence of a seed sequence similar to type I systems (Deveau *et al.*^2008^; Garneau *et al.*^2010^; Sapranauskas *et al.*^2011^; Semenova *et al.*^2011^; Jinek *et al.*^2012^; Martel and Moineau 2014). Further experiments revealed that the PAM is only recognized in a dsDNA context and that cleavage by the HNH and RuvC domains of Cas9 occurs within the protospacer, exactly 3 nt away from the PAM (Garneau *et al.*^2010^; Gasiunas *et al.*^2012^; Jinek *et al.*^2012^; Magadan *et al.*^2012^). It was also shown, that the HNH domain appears to have a fixed cleavage site, while the targeting position for the RuvC domain is defined by a ruler mechanism, in which a linker between the PAM and the protospacer influences the cleavage site, yielding non-blunt-ended cleavage in very rare cases (Chen, Choi and Bailey 2014).

Early observations of the conserved ability to form tracrRNA:crRNA duplexes despite the large diversity in tracrRNA anti-repeat and crRNA repeat sequences indicated coevolution of tracrRNA and crRNA (Deltcheva *et al.*^2011^). Further analysis of phylogenetic trees and sequence variability of tracrRNA anti-repeat, CRISPR repeat and Cas9 orthologs led to the proposal that the dual-tracrRNA:crRNAs have functionally coevolved with the Cas9 proteins (Deltcheva *et al.*^2011^; Jinek *et al.*^2012^; Chylinski, Le Rhun and Charpentier 2013; Chylinski *et al.*^2014^; Fonfara *et al.*^2014^). DNA cleavage by dual-tracrRNA:crRNA-guided Cas9 was reported for Cas9 orthologs from various bacterial species, closely or distantly related to the *S. pyogenes* Cas9 (Jinek *et al.*^2012^; Karvelis *et al.*^2013^; Fonfara *et al.*^2014^). Orthologous Cas9 proteins can utilize non-cognate tracrRNA:crRNAs as guide sequences only when these RNAs originate from loci with highly similar Cas9 sequences (Jinek *et al.*^2012^; Fonfara *et al.*^2014^), demonstrating orthologonality in CRISPR-Cas9 activities. The involvement of a secondary structure of the tracrRNA anti-repeat:crRNA repeat duplex in the specific recognition by Cas9 orthologs was proposed based on similar structure features shared among the exchangeable RNAs (Jinek *et al.*^2012^; Briner *et al.*^2014^; Fonfara *et al.*^2014^).

Although the cleavage complex in type II systems was shown to essentially include three components: tracrRNA, crRNA and Cas9 endonuclease (Jinek *et al.*^2012^), a custom-made single-guide RNA (sgRNA) that combined both tracrRNA and crRNA into one molecule was shown to be capable of complexing with Cas9 to promote effective cleavage of target DNA, paving the way for RNA programmable genome editing using a single guide CRISPR-Cas9 system (Jinek *et al.*^2012^). The sgRNA retains two main characteristics of the natural dual-RNA: a nucleotide sequence at the 5^′^ end that forms specific base pairing with the target DNA and the double-stranded anti-repeat–repeat structure at the 3^′^ end that binds to Cas9. sgRNA-Cas9 forms thus a two-component system in which changes in the guide sequence can program the system to target any DNA sequence of interest owing to the presence of a PAM adjacent to the sequence to be targeted (Jinek *et al.*^2012^). Programmable CRISPR-Cas9 using the *S. pyogenes* system has rapidly and widely been recognized as an effective technology to target, edit or modify the genomes of a large variety of cells and organisms (Doudna and Charpentier 2014; Hsu, Lander and Zhang 2014). The technology was also recently harnessed for programmable RNA recognition and cleavage (O'Connell *et al.*^2014^). In addition to its role in crRNA maturation and interference with DNA, recent studies show Cas9 is also required for the selection of spacers by recognizing the PAM of the protopacers during the phase of adaptation (Heler *et al.*^2015^; Wei, Terns and Terns 2015).

Following the identification of CRISPR-Cas, suggestions indicated that the system could be involved in cellular pathways other than interference with mobile genetic elements (Westra, Buckling and Fineran 2014). The type II-B system of *Francisella novicida* has provided the first evidence for another targeting function of CRISPR-Cas. In this specific case, scaRNA (small CRISPR-Cas associated RNA) is a small RNA that pairs with tracrRNA to form a heteroduplex, similar to the dual-tracrRNA:crRNA (Sampson *et al.*^2013^; Sampson and Weiss 2013). A model was proposed whereby tracrRNA:scaRNA guides Cas9 to target the mRNA of a bacterial lipoprotein. The formation of the targeting complex results in the reduction of lipoprotein production, which in turn enables *F. novicida* to evade the host immune response (Sampson *et al.*^2013^; Sampson and Weiss 2013). Thus, the mRNA-targeting function of the type II-B system via the tracrRNA:scaRNA-Cas9 complex confers to CRISPR-Cas an alternative function in endogenous gene regulation and virulence.

### Composition of type III nucleic acid interference complexes

#### The type III-A DNA/RNA interference complex

Similar to type I CRISPR-Cas, the type III systems are present in a wide range of phylogenetically diverse bacterial and archaeal species (Makarova *et al.*2011a). Both types share significant similarities in the mechanism of crRNA maturation and encode a crRNP interference complex that contains multiple Cas protein subunits with a conserved RNA recognition motif (RRM) fold. The mature crRNAs of type III are primarily processed by the endoribonuclease Cas6 generating the common 8-nt 5^′^ handle. Extensive nucleolytic trimming of the 3^′^ end is observed, producing a pool of two major cellular crRNA species (39–45 nt) with a specific 6-nt length difference (Carte *et al.*^2008^; Hale *et al.*^2009^; Zhang *et al.*^2012^). In contrast to type I and II systems, type III systems do not rely on the presence of a PAM sequence during interference (Staals *et al.*^2014^; Tamulaitis *et al.*^2014^).

The Csm complex (type III-A) from *Su. solfataricus* with a molecular weight of ∼428 kDa is composed of eight different proteins with the stoichiometry of (Csm2)_3_-(Csm3.1)_1_-(Csm3.2)_4_-(Csm3.3)_1_-(Cas10)_1_-(Csm3.4)_1_-(Csm4)_1_-(Csm3.5)_1_. Several Csm3 subunits and one Csm4 subunit form the crRNA-binding backbone (Rouillon *et al.*^2013^). It was suggested that the function of large and small subunits is fulfilled by Cas10 and a trimer of Csm2 proteins based on their location in a Csm complex structure (Rouillon *et al.*^2013^). The purified Csm complex of *Thermus thermophilus* has a nearly identical molecular weight of ∼427 kDa with a slightly differing composition. Here, six Csm3 and two Csm4 subunits form the crRNA-interacting backbone and additionally the protein Csm5 was identified as an integral part of the complex (Staals *et al.*^2014^). This crRNP complex, as well as the Csm complex of *S. thermophilus*, was shown to target complementary ssRNA and cleave it at multiple sites *in vitro* and *in vivo* (Staals *et al.*^2014^; Tamulaitis *et al.*^2014^). In *Staphylococcus epidermidis*, the type III-A system was shown to target plasmid DNA, as well as temperate phages, in a transcription-dependent manner (Marraffini and Sontheimer 2008; Goldberg *et al.*^2014^).

#### The type III-B RNA interference complex

The Cmr complex (type III-B) was analyzed in the bacterium *T. thermophilus* and was shown to have an estimated molecular weight of ∼365 kDa and a complex stoichiometry of (Cmr1)_1_-(Cas10)_1_-(Cmr3)_1_-(Cmr4)_4_-(Cmr5)_3_-(Cmr6)_1_ (Staals *et al.*^2013^). A similar subunit composition was found in *Pyrococcus furiosus* with either three (Spilman *et al.*^2013^) or four Cmr4 subunits (Hale *et al.*^2014^). The crRNP backbone is formed by Cmr3 and multiple copies of Cmr4. The large and small subunits are proposed to be represented by Cas10 and three subunits of Cmr5 (Spilman *et al.*^2013^; Staals *et al.*^2013^). The type III-B systems exclusively target ssRNA and not DNA sequences that are complementary to the crRNA (Hale *et al.*^2009^, ^2012^, ^2014^; Zhang *et al.*^2012^).

## STRUCTURE AND ASSEMBLY OF THE BACTERIAL TYPE I-E DNA INTERFERENCE COMPLEX

A first overview of the general morphology of the I-E Cascade structure at a resolution of 8 Å was obtained by single-particle cryoelectron microscopy in combination with further structural and biochemical studies (Brouns *et al.*^2008^; Jore *et al.*^2011^; Wiedenheft *et al.*2011a). Recently, the crystal structure of different I-E Cascade complexes with resolution between 3.03 and 3.24 Å was solved by several groups, providing important insights into Cascade assembly and the mechanism of target DNA recognition (Jackson *et al.*2014a; Mulepati, Heroux and Bailey 2014; Zhao *et al.*^2014^). Overall, the complex consists of 11 protein subunits ((Cse1)_1_-(Cse2)_2_-(Cas5)_1_-(Cas7)_6_-(Cas6e)_1_) and a single 61-nt crRNA (Fig. 2). The shape of the structure was described to resemble a seahorse (Jore *et al.*^2011^). Accordingly, Cas6e is tightly bound to the 3^′^ stem-loop structure of the mature crRNA and positioned at the head of the complex (Wiedenheft *et al.*2011a). The 5^′^ handle of the crRNA is placed between Cas5 and the large subunit Cse1 at the tail of the seahorse. The head and tail of the crRNA are bridged by six Cas7 copies (Cas7.1–Cas7.6) that form a helical backbone, while the belly is represented by two Cse2 subunits (Jackson *et al.*2014a).

### Interactions between crRNA and Cas6e at the Cascade head

The structure of Cas6e consists of two RRMs that are connected by an eight-residue linker. The typical RRM-fold consists of four anti-parallel beta-strands and two alpha-helices that are arranged in a β1-α1-β2-β3-α2-β4 pattern. The β-strands are ordered in a four-stranded antiparallel β-sheet with two α-helices packed on one side (Ebihara *et al.*^2006^). The two β-sheets face one another and create a funnel-shaped cleft. Cas6e comprises a positively charged basic patch opposite of this cleft that interacts with the crRNA 3^′^ end. The major groove of the crRNA stem loop is wrapped around a positively charged Cas6e groove-loop (i.e. the β6–β7 hairpin, residues 90–119) (Jackson *et al.*2014a; Zhao *et al.*^2014^). This positioning of the crRNA stem loop directs the scissile phosphate group into the active site of Cas6e. Thus, Cas6e recognizes bases on both sides of the stem loop and interacts tightly with the 3^′^ handle of the crRNA after cleavage (Sashital, Jinek and Doudna 2011).

### Structure of the Cas7:crRNA backbone

The prominent backbone of Cascade is assembled via the oligomerization of six Cas7 subunits around the mature crRNA (Fig. 2). The intertwined structure is arranged in six discrete segments, in which one nucleotide is buried, followed by five accessible bases that are coordinated in a pseudo A-form configuration. The typical structure of Cas7 resembles a right hand and consists of distinct regions termed fingers (residues 59–180), a palm (residues 1–58, 181–189 and 224–263) and a thumb (residues 190–223) domain (Mulepati, Heroux and Bailey 2014). The palm contains a modified RRM and two smaller loops inserted in this RRM are forming a web between the thumb and the fingers (Jackson *et al.*2014a). The five crRNA base segments that are accessible for target DNA hybridization are in contact with the palm domain via several conserved polar and positively charged residues (K27, S40, Q42 and K45). Additionally, a conserved M166 residue intercalates with the third and fourth base in each segment, which keeps the nucleotides apart and helps to distort the A-form configuration (Jackson *et al.*2014a; Zhao *et al.*^2014^). In contrast to the classical RRM arrangement, the α1-helix is not located on the back of the β-sheets, but is positioned perpendicular on the central ß-sheet. This helix contains several conserved residues (W199, F200, T201 and V203) that interact with three consecutive phosphates of the crRNA (Jackson *et al.*2014a; Mulepati, Heroux and Bailey 2014). These interactions introduce two successive ∼90° turns in the crRNA backbone, which causes every sixth base in the segment (crRNA positions: 6, 12, 18, 24 and 30) to flip outwards (Jackson *et al.*2014a; Mulepati, Heroux and Bailey 2014; Zhao *et al.*^2014^). This flipped base is further buried between the α1-helix of the palm and the thumb of an adjacent Cas7 subunit. Therefore, the conformations of the Cas7.2–Cas7.6 subunits are identical and the neighboring subunits display two pronounced protein:protein interactions sites next to the crRNA contact (Jackson *et al.*2014a; Mulepati, Heroux and Bailey 2014). A first area (∼1500 Å^2^) of interaction is formed between the thumb-base and the palm back of one Cas7 with the palm front of the adjacent Cas7 subunit. The second area (∼400 Å^2^) of interaction is generated between the thumb-tip and the fingers of neighboring subunits (Mulepati, Heroux and Bailey 2014). The Cas7:crRNA backbone interacts with the Cascade head via protein:protein contacts between Cas6e and Cas7.1 at the 3^′^ end of the crRNA (Fig. 2). In contrast to the other five Cas7 subunits, a short helix located on the thumb-tip of Cas7 (contact residues: W199, F200 and V203) is inserted into the hydrophobic funnel-shaped cleft of Cas6e, which lies opposite of the crRNA interaction site. As a result, the flexible Cas7 thumb domain is rotated outwards by 73° in comparison to the thumb domains of the other five Cas7 subunits (Jackson *et al.*2014a; Mulepati, Heroux and Bailey 2014; Zhao *et al.*^2014^).

### Interactions between crRNA and Cas5 at the Cascade tail

Type I-E mature crRNAs are characterized by a conserved 8-nt 5^′^ handle, generated by Cas6e cleavage within the repeat sequence. The respective nucleotides (numbered from position −8 to −1) are protected by the Cas5 subunit within Cascade (Jore *et al.*^2011^). The Cas5 structure reveals a palm domain that includes a modified RRM (residues 1–78 and 115–224) and a thumb (residues 79–114) domain (Fig. 2). Thus, structural similarities between Cas5 and Cas7 are apparent (Jackson *et al.*2014a; Mulepati, Heroux and Bailey 2014). Additionally, the positioning of the Cas5 thumb supports a function analogous to the Cas7 thumb. The Cas5 thumb folds over the kinked base at position −1, employing similar residues in the binding pocket that Cas7 uses for binding (L89 and T87) and thus ensures the crRNA A-form configuration of the first base of the spacer (Jackson *et al.*2014a; Mulepati, Heroux and Bailey 2014; Zhao *et al.*^2014^). This assembly guarantees the segmentation of the Cas5-protected 5^′^ handle from the accessible crRNA guide region that is clamped by the subunit Cas7.6. The Cas5 thumb contacts the adjacent Cas7.6 subunit at the fingers domain, which leads to a ∼180° rotation of the fingers in comparison to the fingers of the other Cas7 subunits (Jackson *et al.*2014a). Consequently, a broader 28 Å gap appears between the proximate Cas7.5 and Cas7.6 fingers (Jackson *et al.*2014a). The buried crRNA bases of positions −8 to −2 form a hook-like structure and are sandwiched between Cas5 and the web of Cas7.6 via extensive contacts of charged and polar residues. The terminal three bases (A-8, U-7 and A-6) interact with binding pockets on top of the glycine-rich α1-helix of Cas5 with sequence-specific contacts between Y145 and U-7. The three central bases (A-5, A-4, C-3) form a triplet stack orthogonal to A-6 positioned between the palm domains of Cas5 and Cas7.6. The position C-2 forms sequence-specific hydrogen bonds with R108 of the Cas5 thumb (Jackson *et al.*2014a; Mulepati, Heroux and Bailey 2014). Mutation of residue R108 showed that this position is essential for the interference mechanism (Zhao *et al.*^2014^).

### Positioning of small and large subunits in Cascade

The *E. coli* large subunit protein Cse1 has a unique globular fold that contains a zinc-ion coordinated by four cysteine residues (C140, C143, C250 and C253) and a C-terminal four-helix bundle (Mulepati, Orr and Bailey 2012; Jackson *et al.*2014a). The interaction of the large subunit Cse1 with Cas5 and the crRNA 5^′^ handle at the tail is mediated via a short α-helix within a loop (termed L1, residues 130–143) that inserts into a Cas5 helix-binding pore (Fig. 2) (Jackson *et al.*2014a; Mulepati, Heroux and Bailey 2014). This cylindric pore is formed when the Cas5 thumb reaches the Cas7.6 subunit and thereby allows base-specific contacts of Cse1 (residues F129, V130, N131 and Q132) to the accessible A-A-C triplet stack of the crRNA 5^′^ handle. Additional contact sites are observed between the globular domain of Cse1 and the RRM of Cas5 (Jackson *et al.*2014a; Mulepati, Heroux and Bailey 2014). Furthermore, the external α-helix of the four-helix bundle on top of the globular domain interacts with the C-terminal domain of the second small subunit Cse2.2 via salt bridge formation (Cse1 R483:Cse2.2 E150). The two Cse2 subunits that form the Cascade belly are assembled as a head-to-tail dimer, and the Cse2.1 protein interacts with Cas6e (Fig. 2) (Jackson *et al.*2014a; Mulepati, Heroux and Bailey 2014). Several crystal structures of Cse2 were solved that revealed its α-helical bundle scaffold and identified multiple basic patches on the protein surface (Agari *et al.*^2008^; Nam *et al.*2012a). Direct contacts of Cse2 to the crRNA are not observed, but the attachment of both Cse1 and Cse2 to the Cas7 backbone is mediated by five unique binding spots that are all formed by a triad of (i) the negatively charged residue D22 of Cas7, (ii) a positively charged residue of Cse1 and Cse2 (R27 and R101 of Cse2.1 or Cse2.2, as well as K474 of Cse1) and (iii) a stabilizing aromatic side chain of W199 of the following Cas7 subunit (Mulepati, Heroux and Bailey 2014; Zhao *et al.*^2014^). Thus, each Cse2 subunit is connected to three Cas7 subunits (Cse2.1:Cas7.1–3 and Cse2.2:Cas7.3–5) and Cse1 is connected to two Cas7 subunits (Cse1:Cas7.5–6) (Zhao *et al.*^2014^).

### Interaction of Cascade with a target DNA

The crystal structure of the Cascade-crRNA in complex with a 32-nt ssDNA protospacer has facilitated further insights into the DNA targeting mechanism and the localization of the DNA strands within the assembly (Mulepati, Heroux and Bailey 2014). The two strands of the crRNA:target hybrid shape as a ribbon-like structure that does not form a helix, but is underwound due to the crRNA kinks at every Cas7 segment (Mulepati, Heroux and Bailey 2014). Cas7 inhibits the base pairing of complementary nucleotides at the kink, as the thumb of Cas7 extends towards the finger of the adjacent Cas7 subunit passing directly between the strands of the hybrid and shielding the crRNA base. This kink is observed at five crRNA positions in the guide region (position: 6, 12, 18, 24 and 30) due to the linkage of the Cas7 subunits and in one position of the 5^′^ handle due to Cas5 thumb–Cas7 interactions (position: −1) (Mulepati, Heroux and Bailey 2014). Consequently, each 5-bp segment is distorted from a canonical A-form ensuring continuous accessibility of the guide region. The Cas7 subunits are not only interacting with the crRNA backbone via the palm, but are also contacting the target DNA across the minor groove via the thumb-tip (H213 and L214) and the fingers of the adjacent Cas7 (residue 109–111, 163–169) (Mulepati, Heroux and Bailey 2014). Interestingly, the same binding residues that are observed to attach the large (K474) and small subunit (R27 and R101) to the Cas7 backbone are involved in contacting the displaced DNA bases of the target strand. Their function is likely to hold the target strand in position for crRNA hybridization between the Cas7 backbone and Cse1, Cse2.2 and Cse2.1 (Mulepati, Heroux and Bailey 2014).

Insights into the localization of the non-target DNA strand were obtained by cryo-EM studies of Cascade-dsDNA complexes. The PAM-proximal end of the DNA was found to bind between Cas7.5, Cas7.6 and Cse1 (Westra *et al.*2012b; Hochstrasser *et al.*^2014^). The combination of crystal structure and cryo-EM density data revealed potential interactions between the dsDNA and several basic residues (Cas7.5: K137, K138, K141 and Cas7.6: H67, K105) that are exposed in the broader gap of the Cas7.6 and Cas7.5 fingers (Mulepati, Heroux and Bailey 2014). A conserved structural motif accessibly located on the L1 loop of Cse1 was identified that influenced PAM recognition, resulting in destabilization of the target DNA duplex and crRNA-directed strand invasion (Sashital, Wiedenheft and Doudna 2012; Tay, Liu and Yuan 2015). This region is disordered in the Cascade:ssDNA structure, suggesting its flexibility in the absence of dsDNA (Mulepati, Heroux and Bailey 2014). The non-target strand is displaced during crRNA:ssDNA pairing (Jore *et al.*^2011^). A distinctive basic groove spanning from Cse1 via the Cse2 dimer to Cas6e provides a binding site for the displaced strand (Mulepati, Heroux and Bailey 2014; Tay, Liu and Yuan 2015). Mutations in the conserved positive patch of Cse1 from *Thermobifida fusca* abolished the DNA binding (Tay, Liu and Yuan 2015). Mechanistically, after PAM recognition and target pairing, the non-target strand has to loop around the four-helix bundle of Cse1 and is then directed via basic residues of Cse2 (R53, R110, R142, R143) as well as of Cas7 (K34, K299, K301) (Mulepati, Heroux and Bailey 2014). The cryo-EM studies of Cascade bound to dsDNA identified structural rearrangements of the large and small subunit upon DNA targeting (Wiedenheft *et al.*2011a). During DNA binding, the Cse2 dimer moves ∼16 Å relative to Cse1, which leads to a ∼30° rotation of the Cse1 four-helix bundle and a ∼15° rotation of the Cse1 base. These rearrangements make the binding sites of Cse2 within the basic groove accessible for non-target DNA binding and coordinate a platform for Cas3 recruitment (Hochstrasser *et al.*^2014^; Mulepati, Heroux and Bailey 2014).

## STRUCTURES AND FUNCTIONAL DOMAINS OF THE TYPE I SIGNATURE PROTEIN CAS3

**Figure 4.**
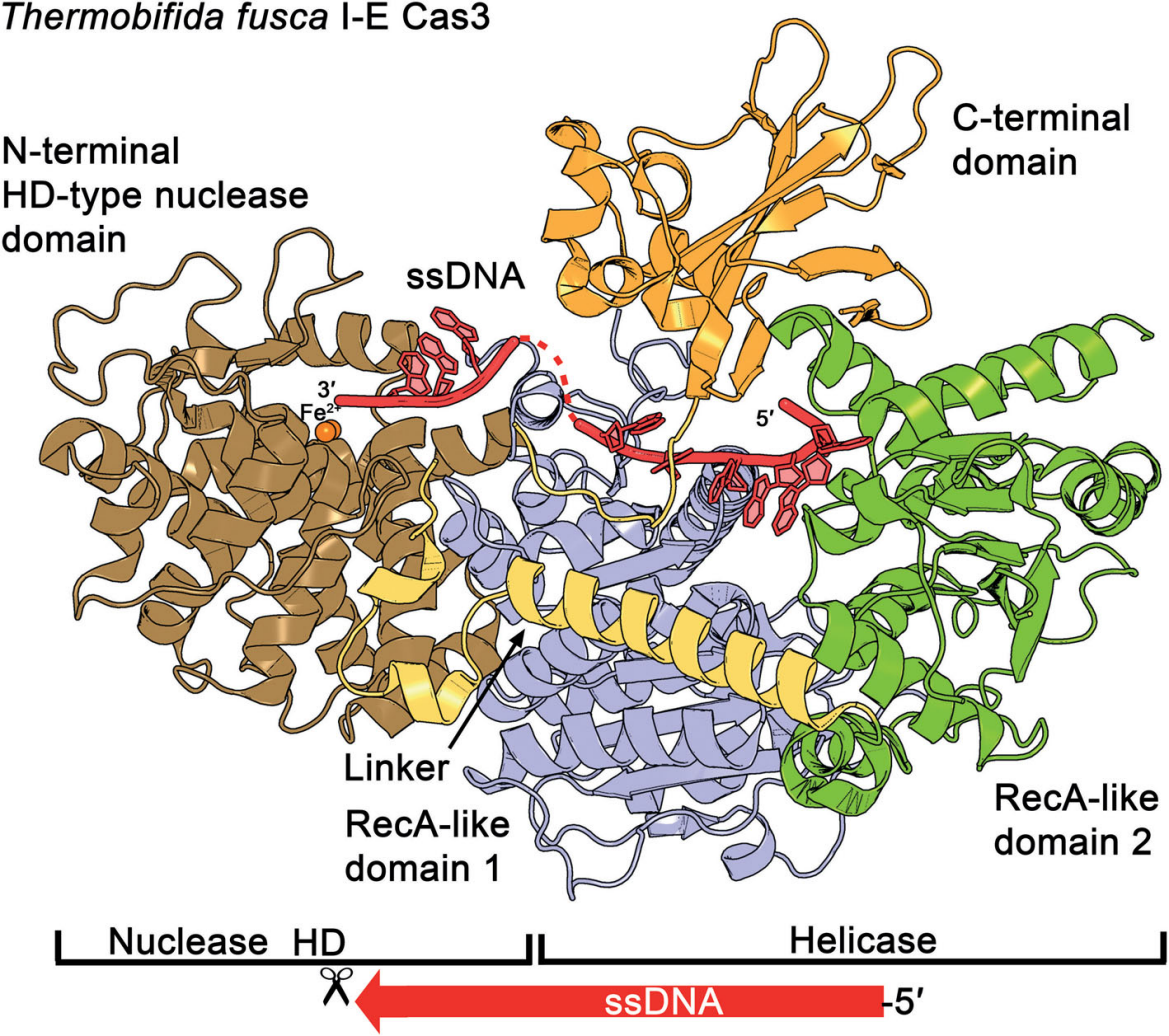
Structure of the DNA nuclease Cas3. The type I-E Cas3 crystal structure of *Th. fusca* (pdb: 4QQW) reveals two tandem RecA-like domains, one HD-type nuclease domain and a CTD located at the top of the ensemble. The core helicase, containing two RecA-like domains, forms a cleft that locates the residues for the binding of NTP, Mg^2+^ ions and the ssDNA substrate. Two Fe(II) ions are located at the catalytic center's HD motif. The 5^′^ end of the ssDNA enters Cas3 from the RecA2 side and is further threaded to RecA1 and the HD-type nuclease domain (indicated by a scissor). The CTD is proposed to close the ssDNA channel and to contact the Cascade complex.

Cas3 is the signature protein of type I CRISPR-Cas systems. This protein plays a crucial role in the viral defense reaction, as it is responsible for target degradation (Brouns *et al.*^2008^). Phylogenetic analyses of Cas3 proteins from all type I systems revealed a common helicase domain core and diverse N-terminal and C-terminal accessory domains (CTD; Jackson *et al.*2014b) (Fig. 4). The core helicase domain shows highly conserved residues of superfamily 2 (SF2) helicases including the NTP-binding Walker A and Walker B motifs (Jansen *et al.*^2002^; Makarova *et al.*^2002^). SF2 helicases contain a tandem RecA-like fold, which forms a cleft coordinating the amino acids responsible for the binding of NTP, Mg^2+^ ions and nucleic acid substrates (Cordin *et al.*^2006^; Fairman-Williams, Guenther and Jankowsky 2010). Biochemical studies show that Cas3 enzymes of type I-E are ATP dependent and unwind duplex DNA in a 3^′^-5^′^ direction via an inchworm-like mechanism (Sinkunas *et al.*^2011^; Mulepati and Bailey 2013). Additionally, all type I systems encode an HD nuclease that is either fused as an N-terminal accessory domain to the helicase core (type I-B, I-C, I-E and I-F) or encoded by a separate gene (type I-A, type I-B and I-D). The HD nuclease is characterized as a metal-dependent exo- and endonuclease in the presence of divalent metals that are coordinated by the active site HD motif (Beloglazova *et al.*^2011^; Mulepati and Bailey 2011; Sinkunas *et al.*^2011^). Typically, a CTD is fused to the helicase core. This domain is suggested to connect Cas3 and Cascade (Gong *et al.*^2014^; Huo *et al.*^2014^). Noteworthy, Cas3 of type I-F is additionally fused to a Cas2-like domain at its N-terminus and interacts with Cas1 (Makarova *et al.*2011a; Richter *et al.*2012a).

In 2011, the crystal structures of the Cas3 nuclease domains from *T. thermophilus* (type I-E) and *Methanocaldococcus jannaschii* (type I-A) were published (Beloglazova *et al.*^2011^; Mulepati and Bailey 2011). More recently, two crystal structures of the entire type I-E Cas3 protein were solved, one from *Thermobaculum terrenum* in complex with ATP, and one from *Th. fusca* in complex with an ssDNA substrate and ATP (Gong *et al.*^2014^; Huo *et al.*^2014^). The Cas3 structure shows the typical arrangement of tandem RecA-like domains and an HD domain docked at RecA1 with the CTD domain located at the top of the helicase core (Fig. 4). The contact sites for the essential cofactors ATP and Mg^2+^ are present within the RecA1 domain, in the cleft towards the RecA2 domain. In *Th. fusca*, the binding of Cas3 to ATP involves the residues Q284 (Q motif) and in addition G308, E309 and G310 (Walker A motif). The Mg^2+^ ion is coordinated via D451 and E452 (DEAH, Walker B motif) (Huo *et al.*^2014^).

All available HD domain structures reveal a globular shape with a concave surface (Mulepati and Bailey 2011). The HD domain:RecA1 contact area in *Th. fusca* is provided via a hydrophobic interface of ∼4200 Å^2^, which includes several conserved residues within the inner concave site (HD: W216, L217, L260 and RecA1: W406, R412, L415, F441, W470) (Huo *et al.*^2014^). Different metal ions were found to be coordinated by the invariant HD residues in the three available HD domain structures. In *Th. fusca*, two Fe(II) ions are positioned by the residues of this HD motif (H83 and D84) and several conserved histidines (H37, H115, H149, H150) (Huo *et al.*^2014^). In contrast, the second type I-E HD domain structure from *T. thermophilus* contained one Ni^2+^ ion in the HD motif (residues: H69, D70 and H24, D205) (Mulepati and Bailey 2011). In the type I-A HD domain structure from *M. jannaschii*, two Ca^2+^ ions were identified to interact with residues of the HD motif (residues: H66, D67 and H91, H123, H124) (Beloglazova *et al.*^2011^). Furthermore, ssDNase activity was observed with several transition-metal ions including Mg^2+^, Mn^2+^, Co^2+^, Cu^2+^ and Zn^2+^ indicating a broad range of functional metal cofactors (Mulepati and Bailey 2011; Sinkunas *et al.*^2011^; Gong *et al.*^2014^; Huo *et al.*^2014^). The positioning of these transition-metal ions in the catalytic center suggests that their role in the cleavage mechanism is to coordinate a deprotonated water molecule for nucleophilic attack of the ssDNA (Huo *et al.*^2014^).

The cocrystallized ssDNA molecule in the *Th. fusca* Cas3 provides further insights into the coordinated path of the target DNA during its degradation (Huo *et al.*^2014^) (Fig. 4). The 5^′^ end of the ssDNA gets incorporated into Cas3 from the RecA2 side via a postulated separation hairpin (position: 715–727) that is conserved in many SF2 helicases and intercalates into the dsDNA. The CTD contacts surface loops of both RecA domains on top and a closed ssDNA channel is formed. The ssDNA is then further threaded from RecA2 to RecA1 by contacts of several salt-bridge and hydrogen-bond interactions (Gong *et al.*^2014^; Huo *et al.*^2014^). Finally, the positioning of the ssDNA in the catalytic center of the HD nuclease is supported by K411 and W216, resulting in a sharp bend of the DNA backbone (Huo *et al.*^2014^).

**Figure 5.**
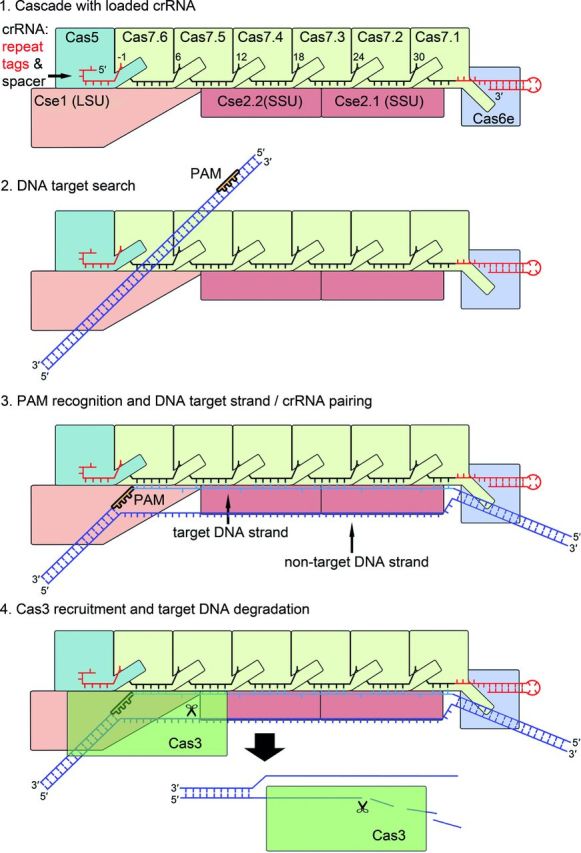
Mechanism of type I Cascade-mediated DNA interference. After the assembly of the crRNA-loaded Cascade, the surveillance complex (SSU: small subunits, LSU large subunit) scans DNA sequences. Potential DNA targets are identified via PAM recognition. This triggers the destabilization of the DNA duplex and allows the crRNA to pair with the target strand, while the non-target strand is displaced and spanned via the large and small subunit. Following R-loop formation, interaction sites at the base of the large subunit enable a stable interaction with Cas3. The HD domain of Cas3 nicks the DNA strand downstream of the PAM and the duplex is further unwound in 3^′^–5^′^ direction and degraded. The remaining single-stranded target DNA is proposed to be cleaved by the stand-alone Cas3 enzyme.

Several biochemical studies revealed that Cascade recruits Cas3 after it formed the crRNA:target DNA hybrid structure, the so-called R-loop (Westra *et al.*2012b; Mulepati and Bailey 2013; Sinkunas *et al.*^2013^). Negative-stain EM visualization of I-E Cascade:dsDNA complexes that were incubated with Cas3 revealed that Cas3 binds between the four-helix bundle and the base of Cse1, a region where conformational changes during DNA binding were observed (Hochstrasser *et al.*^2014^). Mapping this information onto the Cascade crystal structure identified several residues (E192, E280, N376 and T383) and two loops at the Cse1 base (residues 288–294, 318–323) that might mediate the Cas3 interaction (Mulepati, Heroux and Bailey 2014). Additionally, the Cas3 protein of *Th. fusca*, lacking the CTD domain, showed a decreased affinity for Cascade (Huo *et al.*^2014^). These structural insights into the target degradation pathway support a model of concerted events: (i) Cascade assembly, (ii) target search and R-loop formation, (iii) Cas3 recruitment and finally (iv) target DNA cleavage (Fig. 5). In the following section, we review these individual stages as the combination of available biochemical data and the Cascade structures considerably expanded our knowledge of the Cascade-mediated DNA interference mechanism.

## MECHANISM OF CASCADE-MEDIATED DNA INTERFERENCE

### Cascade assembly

Cas6e cleaves pre-crRNA within the repeats at the level of the RNA stem-loop structure and remains tightly bound to the 3^′^ handle, protecting the RNA stem loop from unspecific nucleolytic trimming (Brouns *et al.*^2008^; Sashital, Jinek and Doudna 2011). This initial reaction is proposed to serve as a platform for the coordinated Cascade complex formation (Jore *et al.*^2011^). However, the order of the following Cascade backbone and tail assembly steps is not known. It is possible that Cas5e directly caps the crRNA's 5^′^ handle via base-specific interactions, which leads to the hook-like structure of the crRNA 5^′^ tag (Jackson *et al.*2014a; Mulepati, Heroux and Bailey 2014; Zhao *et al.*^2014^). The structure of Cas5e and Cas7 revealed a conserved palm-thumb domain arrangement that explains the intertwined assembly of the Cascade backbone. The thumb of either Cas5 or each of the six Cas7 subunits kinks the crRNA at position −1 in the 5^′^ handle and every sixth position in the spacer sequence, and buries the base between the thumb and the palm in a positively charged pocket of the adjacent Cas7 subunit (Jackson *et al.*2014a; Mulepati, Heroux and Bailey 2014). Accordingly, the 5-nt segments of the crRNA guide are stretched along the Cas7 palm and are therefore accessible for complementary base pairing (Fineran *et al.*^2014^). The thumb of the subunit Cas7.1 positioned at the end of the crRNA guide then folds into a cleft of Cas6e and links the whole crRNA backbone (Jackson *et al.*2014a; Mulepati, Heroux and Bailey 2014). It remains to be seen if Cas7 oligomerization at the crRNA is either started or stopped at one of the two terminal caps. These steps ensure that the crRNA is fully protected in the cell. The final assembly is suggested to involve the subunits that interact with the DNA. The two small Cse2 subunits are connected to the crRNA backbone via protein:protein interactions between one Cse2 and three Cas7 subunits (Jackson *et al.*2014a; Mulepati, Heroux and Bailey 2014). The large subunit Cse1 is positioned at the Cascade tail and interacts with Cas5, Cas7 and Cse2 (Jackson *et al.*2014a; Mulepati, Heroux and Bailey 2014). This step completes Cascade assembly and ensures the presence of a surveillance complex that constantly screens the cell for potential target DNAs.

### Cascade interactions with PAM sequences

Target DNAs are identified via different quality checkpoints to ensure that only harmful DNA is degraded and that e.g. the host genome is not targeted (van der Oost *et al.*^2014^). First, Cascade scans the dsDNA for a potential PAM sequence (Westra *et al.*2012b; Hochstrasser *et al.*^2014^; Rollins *et al.*^2015^). The PAM is a conserved type-specific short sequence (2–5 nt) that directly flanks the protospacer sequence on the mobile genetic element. PAMs are not inserted in the CRIPSR array during the stage of spacer acquisition. This specific signature of the invading DNA allows the interference complex to differentiate between the spacer of the CRISPR locus on the host chromosome (self) and the invading DNA (non-self) (Deveau *et al.*^2008^; Mojica *et al.*^2009^; Shah *et al.*^2013^). Thus, PAM sequences of the invading DNA do not base pair with the respective positions of the CRISPR repeat flanking the corresponding spacer sequence in the array (Westra *et al.*^2013^). Initially, the PAM is used for the selection of new spacers during acquisition to guarantee the integration of functional spacers in the host CRISPR locus (Datsenko *et al.*^2012^; Swarts *et al.*^2012^; Yosef, Goren and Qimron 2012). Since spacer acquisition and DNA interference are performed by two different molecular machineries, analyses showed that the respective motifs are not necessarily identical for these two processes. The PAM recognition is an exact process during the DNA interference reaction, as typically only a single 2–3 bp motif is tolerated (Westra *et al.*^2013^; Rollins *et al.*^2015^). One exception is the type I-B system of *Haloferax volcanii*, in which different PAM sequences were found to be functional in DNA targeting (Fischer *et al.*^2012^). Usually, several different PAM sequences of 2–5 bp length are tolerated during the spacer acquisition process (Yosef, Goren and Qimron 2012; Fineran *et al.*^2014^). Therefore, the additional terms spacer acquisition motif and target interference motif for the respective recognition sites were proposed (Shah *et al.*^2013^). In type I systems, the PAM is located on the target crRNA strand at the 3^′^ end of the protospacer (Mojica *et al.*^2009^; Westra *et al.*^2013^). For type I-E, the dsDNA enters Cascade in the gap between Cas7.5 and Cas7.6 and is then transferred to the large subunit Cse1, which has several, mostly non-specific, interactions with the target dsDNA. It is shown that a conserved structural motif of the L1 loop in Cse1 is mediating the PAM identification (Sashital, Wiedenheft and Doudna 2012; Hochstrasser *et al.*^2014^; Mulepati, Heroux and Bailey 2014; Tay, Liu and Yuan 2015).

### Target DNA binding and R-loop formation

The PAM recognition by Cse1 triggers the destabilization of the adjacent DNA duplex and allows the crRNA to access the target DNA strand (Szczelkun *et al.*^2014^). Effective R-loop formation requires the full complementarity of a crRNA seed region and the protospacer, while a mismatch inhibits Cascade-mediated targeting. This seed region covers the positions 1–5 and 7–8 at the 5^′^ end of the crRNA guide adjacent to the PAM (Semenova *et al.*^2011^; Wiedenheft *et al.*2011b; Fineran *et al.*^2014^). The intertwined architecture of the Cascade backbone explains this phenomenon, as the Cas7 and Cas5 folding shields the crRNA base at positions −1 and 6. This makes the adjacent 5-bp segment accessible for target hybridization (Jackson *et al.*2014a; Mulepati, Heroux and Bailey 2014; Zhao *et al.*^2014^). The crRNA-guided strand invasion proceeds further throughout the entire guide sequence, in which the later positions affect primarily the R-loop stability and a limited number of single mismatches between crRNA and ssDNA target are tolerated (Semenova *et al.*^2011^; Fineran *et al.*^2014^; Szczelkun *et al.*^2014^). The non-target strand is displaced during R-loop formation and is spanned from Cse1 and the Cse2 dimer to Cas6e via a basic groove (Tay, Liu and Yuan 2015). At the same time, the large and small subunits rotate upon targeting, which creates binding pockets for R-loop stabilization accessible on the surfaces of Cse2 (Westra *et al.*2012a; Mulepati, Heroux and Bailey 2014). Furthermore, a locking mechanism of the established R-loop structure after the recruitment of Cas3 to Cascade was shown (Rutkauskas *et al.*^2015^). The base pairing of the later crRNA guide nucleotides (positions 24–30), that are accessible upon Cse2.1 movement, might prevent this subunit from retraction (Mulepati, Heroux and Bailey 2014; Szczelkun *et al.*^2014^).

### Cascade-mediated Cas3 recruitment and target DNA degradation

Following R-loop formation and the conformational changes of the large and small subunit, interaction sites at the base of Cse1 are accessible for creating a stable interaction to the CTD of Cas3 (Westra *et al.*2012b; Hochstrasser *et al.*^2014^; Mulepati, Heroux and Bailey 2014). This recruitment at the dsDNA fork site of the R-loop might trigger the CTD to transiently dissociate from the Cas3 core to open the ssDNA channel localized within the helicase domains RecA2 and RecA. After CTD repositioning and closing of the DNA channel, the non-target strand is then positioned in the catalytic center of the HD nuclease domain (Gong *et al.*^2014^; Huo *et al.*^2014^). The HD nuclease nicks the non-target DNA strand at position ∼11–15 downstream of the PAM with the use of two catalytic transition-state metal ions (Mulepati and Bailey 2013; Sinkunas *et al.*^2013^). This nick triggers a change of conformation of the helicase into an active stage, which allows ATP binding and hydrolysis (Sinkunas *et al.*^2011^). The dsDNA is unwound in 3^′^-5^′^ direction at a separation hairpin of RecA2. The movement of the helicase domain translocates the HD domain to a new substrate position for exonucleolytical degradation of the non-target strand (Gong *et al.*^2014^; Huo *et al.*^2014^). It is not known if Cascade is still involved in the dsDNA unwinding or if it is displaced by the progression of the Cas3 helicase (Hochstrasser *et al.*^2014^). The remaining single-stranded target DNA is then exonucleolytically degraded by a stand-alone Cas3 enzyme (Mulepati and Bailey 2013; Sinkunas *et al.*^2013^). Thus, the target DNA is effectively removed from the host cell and the Cascade crRNP can be recycled for another round of target DNA recognition.

## MECHANISM OF TYPE II DNA INTERFERENCE

Three crystal structures (2.2–2.6 Å) of *S. pyogenes* Cas9, one as apoenzyme (Jinek *et al.*^2014^) or two as Cas9 bound to an sgRNA and target DNA (Anders *et al.*^2014^; Nishimasu *et al.*^2014^) were recently resolved. A fourth structure of Cas9 from *Actinomyces naeslundii* was also reported (Jinek *et al.*^2014^). The three studies independently show a bilobal structure of Cas9 proteins in a crescent-shaped conformation (100 × 100 × 50 Å) (Fig. 3).

In *S. pyogenes*, a Cas9 recognition lobe (REC, residues 60–718) consists mainly of α-helices and is involved in both target recognition and binding. The REC lobe is composed of three regions: (i) a long α-helix (bridge-helix, residues 60–94), (ii) a Rec1 domain (residues 94–180, 308–718) and (iii) a Rec2 domain (residues 180–308). REC was reported to be a specific feature of Cas9 proteins since no structural similarities with other proteins could be identified. This lobe is also the least conserved portion of the protein. While the Rec2 domain is dispensable for target cleavage, the Rec1 domain contains a region that is crucial for the recognition of the tracrRNA anti-repeat:crRNA repeat duplex and is therefore necessary for Cas9 activity. Additionally, the Cas9 crystal structure shows that the Rec1 domain and the arginine-rich motif (RRM) on the bridge-helix bind the guide sequence of the sgRNA (Nishimasu *et al.*^2014^). Both motifs interact with the backbone phosphates of the sequence and not with the nucleobases, indicating that recognition of the guide portion of the sgRNA is sequence independent. The recognition of the tracrRNA anti-repeat:crRNA repeat duplex by the REC lobe on the other hand is sequence specific since Cas9 directly interacts with the respective nucleobases. The Rec1 and RRM interaction with the phosphate backbone of the guide RNA leads to an exposure of the eight PAM proximal nucleotides to the solvent. This observation led to the visualization of a seed region, which serves as a nucleation start site enabling target binding and finally cleavage by the nuclease lobe (Jinek *et al.*^2012^). Furthermore, the RRM motif, involved in guide RNA recognition and RNA strand invasion, is a feature that is conserved amongst Cas9 proteins of all three subtypes of type II systems, and is one of the two linkers that connect the two lobes of Cas9 (Nishimasu *et al.*^2014^).

**Figure 6.**
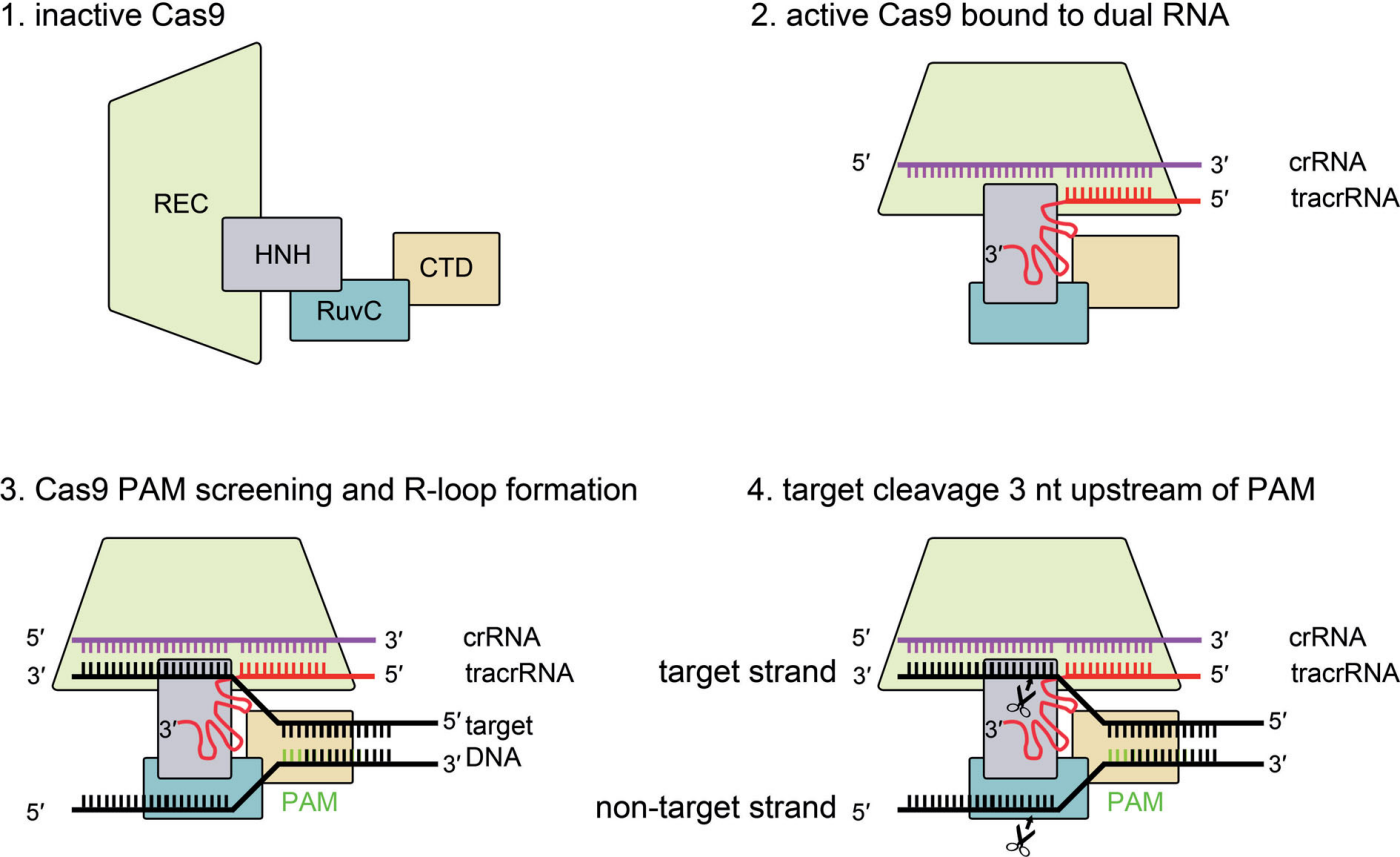
Mechanism of type II Cas9-mediated DNA interference. In the absence of type II specific dual-RNA, Cas9 is in an inactive state. Upon binding to tracrRNA and crRNA, Cas9 undergoes conformational changes, subsequently enabling dual-RNA guided binding to the target DNA. After successful DNA interrogation for a PAM and subsequent nucleation, the guide RNA pairs with the seed sequence on the DNA. This is followed by activation of the Cas9 nuclease domains HNH and RuvC for cleavage of the target and non-target DNA strand, respectively.

The Cas9 nuclease lobe (NUC) is composed of the HNH (residues 775–909) and the split RuvC (residues 1–60, 718–775, 909–1099) nuclease domains and additionally contains a C-terminal topoisomerase homology domain (CTD, residues 1099–1368). The NUC lobe is involved in PAM recognition by the CTD and target cleavage by the domains RuvC and HNH (Anders *et al.*^2014^; Jinek *et al.*^2014^; Nishimasu *et al.*^2014^). Interestingly, it was observed that the HNH domain is disordered in the apoenzyme structure leading to the assumption that this domain is flexible with respect to its position during target DNA recognition and cleavage (Jinek *et al.*^2014^). The target bound structures confirm this hypothesis and show that the HNH domain undergoes a major conformational change yielding an active state of the Cas9 protein (Fig. 6). During this conformational reorientation, the REC lobe also alters its position leading to the formation of a positively charged central channel that harbors the substrate DNA (Anders *et al.*^2014^; Nishimasu *et al.*^2014^). A second but significantly smaller positively charged cleft is situated directly within the NUC lobe. This surface is formed between the CTD and the RuvC domain and harbors the 3^′^ tail of the sgRNA (Jinek *et al.*^2014^; Nishimasu *et al.*^2014^). The elongated CTD (or PAM interacting) is composed of seven α-helices, a three-stranded, a five-stranded and a two-stranded β-sheet. It displays another Cas9-specific fold that lacks structural similarities to other proteins. This part of Cas9 alone recognizes the PAM and it was shown to be sufficient to change this domain to alter PAM specificities (Anders *et al.*^2014^; Nishimasu *et al.*^2014^).

### DNA interrogation and PAM recognition by Cas9:sgRNA

The binding of tracrRNA to crRNA, the subsequent duplex formation and co-processing of the dual-RNA converts an inactive single Cas9 into a DNA surveillance active complex (Deltcheva *et al.*^2011^; Jinek *et al.*^2012^). Once activated, Cas9 is ready to screen for targeted sites on an invading DNA. In type II systems, as well as in type I systems, the discrimination of target and non-target DNA is mediated via the recognition of the PAM (Deveau *et al.*^2008^; Garneau *et al.*^2010^; Sapranauskas *et al.*^2011^). It was shown that binding to target DNA, as well as the subsequent cleavage, requires a PAM (Gasiunas *et al.*^2012^; Jinek *et al.*^2012^; Sternberg *et al.*^2014^). The PAM screening process by Cas9 was investigated in single molecule experiments using DNA curtains. In these experiments, Cas9 displayed unspecific binding to the target, which strongly correlated with the presence of a PAM. Substrates without a PAM but containing the sgRNA-targeted sequences are ignored by Cas9, confirming the importance of the motif (Sternberg *et al.*^2014^). Once Cas9 has identified the dsDNA PAM, it binds to the target. This binding results in a local melting of the target DNA and enables the formation of a RNA-DNA heteroduplex that begins at the PAM (Sternberg *et al.*^2014^). PAM recognition is facilitated by the CTD of Cas9 proteins. In the case of *S. pyogenes* Cas9 (SpyCas9), the 5^′^-NGG-3^′^ PAM is recognized by two arginine residues (R1333 and R1335) that reside in a DRKRY motif that is conserved amongst the 5^′^-NGG-3^′^ recognizing Cas9 proteins (Anders *et al.*^2014^). Interestingly, arginine residues typically pair with guanine nucleotides, while for example glutamine residues usually pair with adenines. Cas9 proteins that recognize the 5^′^-NAAAA-3^′^ PAM contain a QLQ motif instead of the RKR of SpyCas9 (Anders *et al.*^2014^). The recognition of the dG2 and dG3 on the non-complementary (non-target) strand of the target DNA correlates with strand separation since the following interaction of the lock loop (Lys1107-Ser1109) results in a rotation of the first phosphate. This rotation enables pairing with the guide RNA and displays the start of guide RNA and target DNA binding (Anders *et al.*^2014^).

The following R-loop formation is a unidirectional kinetic event in which the PAM is only required for licensing of the DNA distortion but not for R-loop stability. Following the melting event, the guide RNA searches for homology within the 8 – 12 nt seed sequence and H-bonding is formed between the guide RNA and the target DNA sequence (Sternberg *et al.*^2014^; Szczelkun *et al.*^2014^). Only when the seed sequence can pair, the R-loop will continue to propagate from here on (Szczelkun *et al.*^2014^). While Cascade of type I locks the R-loop for Cas3 recruitment, no such stabilization is needed for type II as Cas9 itself is the nuclease cleaving the target. Despite the locking step that is required for type I R-loop formation, the overall kinetics are faster compared to the Cas9 driven event (Szczelkun *et al.*^2014^). In addition to the discrimination between self and non-self, it is likely that binding to the PAM activates the nuclease activity of Cas9 (Sternberg *et al.*^2014^). The analysis of the Cas9 structure bound to its target DNA could show that van der Waals interactions with the C2^′^ atoms of the target help to discriminate between DNA and RNA (Nishimasu *et al.*^2014^).

### Cas9-mediated DNA cleavage

DNA targeting of type II CRISPR-Cas systems requires tracrRNA and crRNA, which can be combined into a single molecule (Jinek *et al.*^2012^). The tracrRNA of *S. pyogenes* contains three regions resulting in stem-loop hairpins located 3^′^ to the tracrRNA:crRNA duplex-forming segment (Deltcheva *et al.*^2011^). Truncations of the wild-type tracrRNA directed at identifying minimal requirements for target DNA binding and cleavage showed that a substantially shortened tracrRNA retaining only 25 nucleotides but comprising the first hairpin could still support cleavage (Jinek *et al.*^2012^). As would be expected, longer sequences more closely resembling the wild type were effective (Jinek *et al.*^2012^), and the second and third loops have been confirmed to enhance stability, while the first loop is indispensable (Nishimasu *et al.*^2014^). The formation of a ternary complex occurs upon target DNA binding. In this event, the sgRNA and the dsDNA target form a T-shaped structure, which yields a four-way junction in which the heteroduplex of guide and target strand is placed into the major groove formed by the REC lobe and the NUC lobe. The double-stranded PAM is located within the C-terminal domain enabling target recognition and R-loop initiation (Anders *et al.*^2014^).

The following target cleavage by Cas9 proteins is performed by the NUC lobe. The two nuclease domains HNH and RuvC cleave the complementary and the non-complementary strand, respectively (Sapranauskas *et al.*^2011^; Gasiunas *et al.*^2012^; Jinek *et al.*^2012^). During this reaction, a double-strand break is introduced within the protospacer, exactly 3 nt upstream from the PAM (Gasiunas *et al.*^2012^; Jinek *et al.*^2012^). With a ββα-fold (or ββα-metal motif) that resembles the active site of T4 endonuclease, the HNH domain employs a single metal mechanism with one Mg^2+^ coordinated by an aspartate and an asparagine residue. An histidine residue serves as general base in the cleavage reaction of the complementary strand and completes the active site of the HNH domain. The RuvC domain is characterized by an RNase H-fold in which two aspartates, a glutamate and an histidine residue coordinate Mg^2+^ ions to facilitate a two-metal reaction to cleave the non-complementary strand of the target DNA (Jinek *et al.*^2014^; Nishimasu *et al.*^2014^). The cleavage activity of both domains yields a blunt-ended double-strand break (Sapranauskas *et al.*^2011^; Gasiunas *et al.*^2012^; Jinek *et al.*^2012^). Following cleavage, Cas9 remains tightly associated to the ends of the targeted DNA, characterizing the protein as a single turnover enzyme (Sternberg *et al.*^2014^).

## STRUCTURES AND TARGETING MECHANISMS OF TYPE III DNA AND RNA INTERFERENCE COMPLEXES

### The Csm complex

The structures of several type III crRNP complexes were recently visualized by electron microscopy, enabling the identification of striking similarities to the Cascade I-E structure (Zhang *et al.*^2012^; Rouillon *et al.*^2013^; Spilman *et al.*^2013^; Staals *et al.*^2013^, ^2014^).

**Figure 7.**
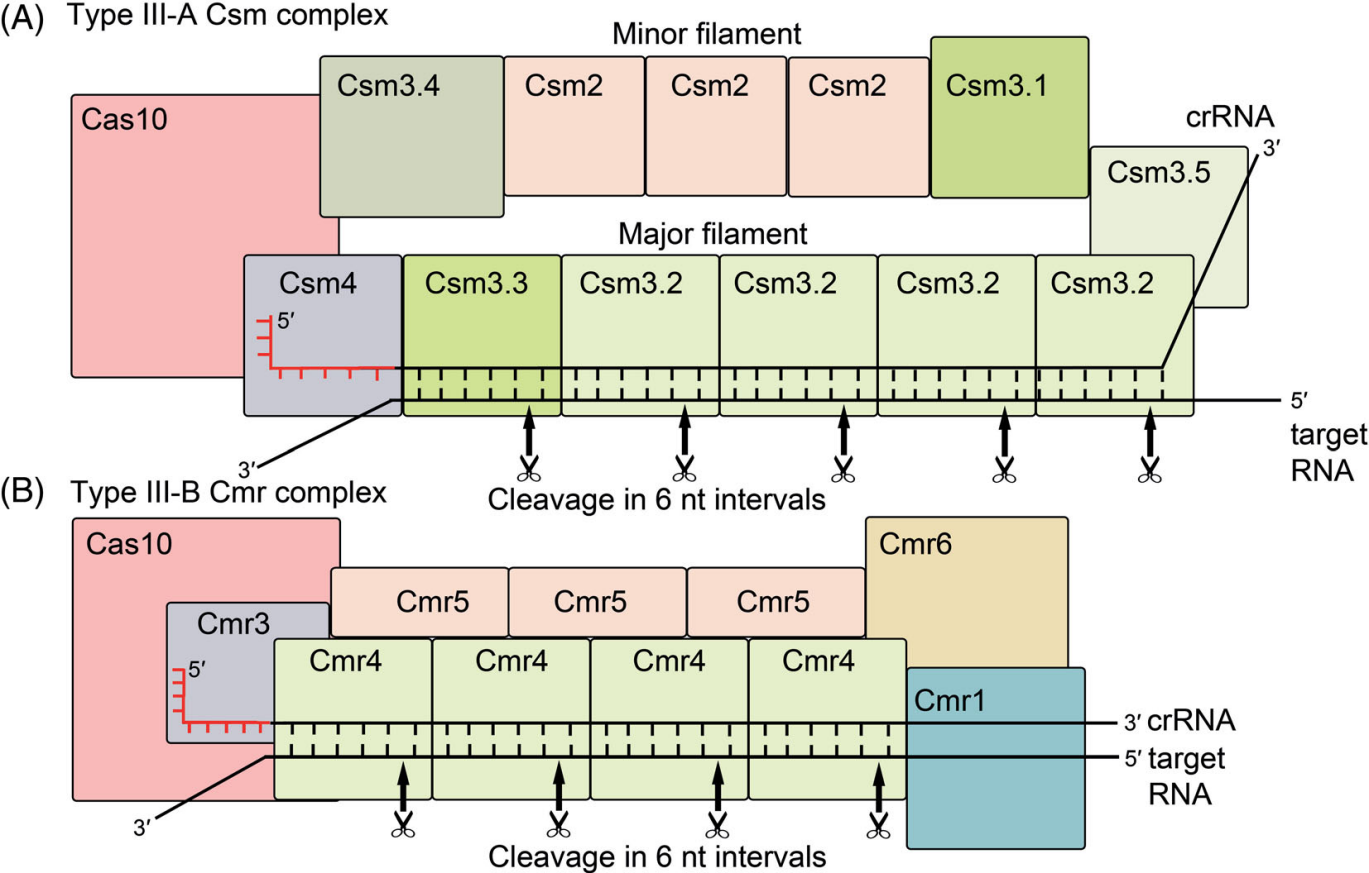
Comparison of type III crRNP-mediated RNA interference. (**A)** The type III-A Csm complex of *Su. solfataricus* is composed of 13 subunits that are arranged as a basal body and two intertwined filaments. Located at the base of the Csm complex is the large subunit Cas10, serving as an anchor for the major and the minor filament. The major filament consists of Csm4 and six Csm3 subunits and binds the crRNA. The minor filament consists of three Csm2 subunits and two additional Csm3 subunits. The Csm3 units that form the backbone of the Csm complex were shown to act as target RNA nucleases (indicated by a scissor) in *S. thermophilus*. (**B)** The type III-B Cmr complex of *P. furiosus* contains an extended helical backbone composed of Cmr4 and Cmr5 subunits. The tail is composed of the stable heterodimer Cas10 and Cmr3, while the curled head contains Cmr1 and Cmr6. The crRNA-binding backbone is formed by several Cmr4 subunits, while the Cas10-Cmr3 heterodimer plays a role in the recognition of the crRNA 5^′^ handle. A second helical structure is formed by three Cmr5 subunits and is inserted alongside the helical Cmr4 filament. Cleavage of target RNA by Cmr4 was observed in 6-nt intervals (indicated by a scissor).

The type III-A Csm complex of *Su. solfataricus* is composed of 13 subunits that are arranged in a basal body and two intertwined filaments (Fig. 7). Several Csm3 paralogs were identified and numbered Csm3.1–Csm3.5 based on their position in the *csm* operon. Located at the base of the Csm complex, the large subunit Cas10 serves as an anchor for the major and the minor filaments (Rouillon *et al.*^2013^). The major filament consists of four Csm3.2 subunits, two different Csm3 subunits (Csm3.5 and Csm3.3) and Csm4. The crRNA is located within this helical assembly (Rouillon *et al.*^2013^). The minor filament consists of three Csm2 subunits and two additional Csm3 subunits (Csm3.1 and Csm3.4). Directly adjacent to the large subunit Cas10 at the base are Csm4 and Csm3.4, while the head of the complex is formed by three Csm3 subunits (Csm3.1, Csm3.2 and Csm3.5). The EM map comparison of the Csm complex and Cascade revealed that the assembly of the Cascade backbone with its six Cas7 subunits correlates with the Csm3 subunit composition of the major filament (Csm3.3, 4 monomers of Csm3.2 and Csm3.5) with an identical pitch of both backbones (Rouillon *et al.*^2013^). In agreement, the crystal structure of the *Methanopyrus kandleri* Csm3 provided evidence that its domain architecture resembles the one of Cas7 (Hrle *et al.*^2013^). As mentioned earlier, Csm4 is located at the base of the Csm complex and its position and overall structure correlates with Cas5 within the Cascade structure (Rouillon *et al.*^2013^). The type III-A crRNPs show some variability in the composition of subunits, as one Csm3 subunit in the minor filament can be replaced by the similar RRM-fold protein Csm5 in *T. thermophilus* or *S. thermophilus*. Overall, five to ten Csm3 and one or two Csm4 subunits, depending on the crRNA length, form the crRNA-interacting backbone (Staals *et al.*^2014^; Tamulaitis *et al.*^2014^).

Similar to type I systems, Cas6 endonuclease cleavage in type III-A generates crRNAs with an 8-nt 5^′^ handle derived from the repeat sequence (Hatoum-Aslan, Maniv and Marraffini 2011; Reeks *et al.*2013b; Shao and Li 2013). This handle might directly be protected by Csm4 and the large subunit Cas10 (Staals *et al.*^2014^; Tamulaitis *et al.*^2014^; Numata *et al.*^2015^). Remarkably, a distinctively shortened repeat-derived crRNA 3^′^ handle was observed that might correspond to a defined protection of the crRNA based on the fixed number of Csm3 subunits in the backbone, while the unprotected RNA overhangs are accessible for exonucleolytical trimming (Hatoum-Aslan *et al.*^2013^; Rouillon *et al.*^2013^; Staals *et al.*^2014^). Thus, variations in the number of Csm3 subunits and the trimming of crRNA 3^′^ handles might be consequences of different spacer lengths or a heterogeneous pool of assembled Csm complexes (Staals *et al.*^2014^; Tamulaitis *et al.*^2014^). Consequently, a stabilizing hairpin structure of the crRNA 3^′^ handle or a stable interaction of Cas6 with the Csm complex was not observed (Rouillon *et al.*^2013^). A requirement for PAM recognition during the targeting of nucleic acid in III-A systems appears to be absent (Staals *et al.*^2014^; Tamulaitis *et al.*^2014^). Instead, the base pairing of the crRNA 5^′^ handle with the host chromosome seems to block the propagation of the interference reaction leading to ‘self-inactivation’ (Marraffini and Sontheimer 2010). The large subunit of the Csm complex is proposed to be involved in targeting, followed by a complementary binding of the crRNA and the matching nucleic acid (Hatoum-Aslan *et al.*^2014^). The minor filament includes the putative small subunit Csm2 and is proposed to be involved in target binding based on the morphological architecture of the two intertwined filaments and the structural similarities between Csm2 and the small subunits of the type I system (Reeks *et al.*2013a; Rouillon *et al.*^2013^). Recently, the binding and cleavage of complementary target ssRNAs was observed *in vivo* and *in vitro*. The Csm complexes generated multiple cuts at 6-nt intervals within the protospacer RNA (Staals *et al.*^2014^; Tamulaitis *et al.*^2014^). The multicopy subunit Csm3, which forms the backbone of the Csm complex, acts as endoribonuclease and thus a mutation of D33 in *S. thermophilus* abolished the RNA cleavage (Tamulaitis *et al.*^2014^). In contrast, the previous reported activity on target DNAs could not be shown *in vitro* yet. Two possible scenarios for DNA degradation are discussed. One candidate for DNA cleavage is Cas10 as it has a permuted HD domain and showed 3^′^-5^′^ exonuclease activity for ssDNA and RNA *in vitro* (Cocozaki *et al.*^2012^; Ramia *et al.*2014a; Jung *et al.*^2015^). Another possibility is that the Csm6 protein needs to be recruited to the Csm complex, as a homologous protein was shown to be involved in the interference mechanism of the III-B system (Deng *et al.*^2013^). A transcription-dependent DNA targeting mechanism was also proposed by Goldberg *et al.*, which may provide a possible explanation for RNA cleavage observed in *in vitro* studies (Goldberg *et al.*^2014^; Staals *et al.*^2014^; Tamulaitis *et al.*^2014^).

### The Cmr complex

Detailed structural information of the RNA-targeting type III-B Cmr complex is available for *T. thermophilus* and *P. furiosus* (Spilman *et al.*^2013^; Staals *et al.*^2013^; Benda *et al.*^2014^). The *T. thermophilus* Cmr complex is composed of 11 subunits with four copies of Cmr4 and three Cmr5 subunits, while in *P. furiosus* either three or four Cmr4 subunits were detected (Spilman *et al.*^2013^; Staals *et al.*^2013^; Benda *et al.*^2014^; Ramia *et al.*2014a). In all cases, two major crRNA species (*T. thermophilus*: 40 and 46 nt or *P. furiosus*: 39 and 45 nt) were identified that were associated with the complex and contained the characteristic 8-nt 5^′^ handle and a trimmed 3^′^ end (Juranek *et al.*^2012^; Staals *et al.*^2013^). In reference to the Cascade sea-horse structure, the Cmr complex was described as a ‘sea-worm’ structure with an extended helical backbone consisting of Cmr4 and Cmr5 subunits (Fig. 7). The tail is composed of the stable heterodimer Cas10 and Cmr3, while the curled head contains Cmr1 and Cmr6 (Spilman *et al.*^2013^; Staals *et al.*^2013^; Benda *et al.*^2014^). Structural analysis of Cas10 revealed a triangular four-domain protein core that has two adenylyl cyclase-like domains arranged as a homodimer and two smaller α-helical domains (Cocozaki *et al.*^2012^). A characteristic treble-clef zinc-finger motif and a degenerated GGDD motif for binding of a Mn^2+^ ion were identified. Additionally, an N-terminal, permuted HD domain, containing two Mn^2+^ ions, is fused to Cas10 but showed no nuclease activity (Cocozaki *et al.*^2012^; Benda *et al.*^2014^). The N-terminal triangular side of Cas10 binds Cmr3, a subunit with two RRM domains (Osawa, Inanaga and Numata 2013; Shao *et al.*^2013^; Benda *et al.*^2014^). At this interface of Cas10-Cmr3, a cleft is formed that serves as a binding pocket for nucleotides embedded within the conserved adenylyl cyclase domain of Cas10. These embedded nucleotides play a role in the recognition of the crRNA 5^′^ handle (Osawa, Inanaga and Numata 2013; Shao *et al.*^2013^; Hale *et al.*^2014^). A helical filament of four Cmr4 subunits closely interacts with the Cas10-Cmr3 base. The Cmr4 protein contains one central RRM core with several inserted elements and a flexible thumb domain, which results in a more elongated structure in comparison to several other members of the Cas7 family (Benda *et al.*^2014^; Ramia *et al.*2014a). The Cmr4 helical structure is capped by Cmr6 whose single RRM domain shows a remarkable similarity to the N-terminal RRM of Cmr1, while the Cmr6 thumb domain superimposes with the thumb domain of Cmr4 (Benda *et al.*^2014^). The last protein in this sequential RRM arrangement of the Cmr backbone is Cmr1. Cmr1 is composed of two close-fitting RRM domains that form a groove of conserved basic and hydrophobic residues (including W38, W39, R154 in *P. furiosus*) for RNA binding of the crRNA 3^′^ region (Benda *et al.*^2014^; Hale *et al.*^2014^; Sun *et al.*^2014^). A second helical structure is formed by three Cmr5 subunits and inserted alongside the helical Cmr4 filament (Benda *et al.*^2014^). Cmr5 is a globular α-helical protein, in which the adjacent subunits interact in a head-to-tail arrangement forming the double-helical Cmr complex body (Park *et al.*^2013^; Benda *et al.*^2014^).

The postulated mechanisms of Cmr complex formation and RNA targeting highlight remarkable similarities to the previously described type III-A system. These encompass Cas6-mediated crRNA maturation and the direct protection of the 5^′^ handle by the Cas10-Cmr3 heterodimer (Spilman *et al.*^2013^; Hale *et al.*^2014^). The crRNA-binding backbone is then assembled by several Cmr4 subunits and the crRNA 3^′^ end is sandwiched between the Cmr1 and the Cmr6 subunit (Benda *et al.*^2014^; Hale *et al.*^2014^). The variable number of three or four Cmr4 subunits observed for different organisms correlates with the two incorporated crRNA species that differ in length by 6 nt (Benda *et al.*^2014^). The assembled Cmr4 backbone supports target interaction via direct contacts of the Cmr4 palm domain to the ssRNA target (Ramia *et al.*2014a). Cas10 is proposed to fulfill the role of the large subunit and, together with the three copies of the small subunit Cmr5, might be involved in complex stability and RNA targeting (Spilman *et al.*^2013^; Staals *et al.*^2013^). The observed target ssRNA cleavage mechanism occurs in three to four 6-nt intervals. The first cut was shown 5 nt away from the 5 ^′^ end and the last cut 14 nt away from the 3^′^ end of the target ssRNA/crRNA duplex (Hale *et al.*^2009^, ^2014^; Staals *et al.*^2013^; Zhu and Ye 2015). Modeling a target ssRNA into the pseudoatomic Cmr complex model identified a potential catalytic center (H15, D26 and E227) within the Cmr4 subunits, whose distances would be able to explain the observed cleavage pattern (Benda *et al.*^2014^). Accordingly, the mutation of the D26 residue abolished endonucleolytical activity without a loss of crRNA interaction or Cmr complex formation (Benda *et al.*^2014^; Hale *et al.*^2014^; Ramia *et al.*2014a). In addition, the Cmr4 residue K46 was identified to be important for crRNA binding (Ramia *et al.*2014a).

## COMMON THEMES AMONG CRISPR-CAS SURVEILLANCE COMPLEXES

The recent elucidation of the structures of several Cas proteins and crRNP complexes, together with biochemical studies, revealed a common architectural core of type I and type III CRISPR-Cas surveillance complexes. Individual subunits often fulfill similar functions, despite their remarkable sequence diversity. Basically, each of these crRNP complexes is built up by a crRNA-binding helical backbone that is composed of at least seven RRM domain proteins, one large subunit and two or three small subunits. The protein backbone responsible for crRNA recognition and interaction is structurally more conserved than the small and large subunits that display more divergent architectures. In the following section, we focus on the comparison of common themes among crRNP surveillance complexes.

### Structural conservation of crRNA binding in type I and type III crRNP complexes

All of the identified proteins that interact with the crRNAs in type I and type III CRISPR-Cas systems contain the RRM domain, an ssRNA-binding domain that is widely distributed in all domains of life and involved in many aspects of RNA binding, regulation and maintenance (Maris, Dominguez and Allain 2005). This group of Cas proteins that contain a conserved RRM domain was previously classified as the RAMP (repeat associated mysterious proteins) superfamily of Cas proteins and further divided into three main groups: Cas7, Cas5 and Cas6 (Makarova *et al.*2011a; Koonin and Makarova 2013). The observed structural similarities support a shared evolutionary origin of all crRNA-interacting Cas proteins. Subsequently, Cas proteins evolved with specialized functions of their individual RRM domains (Koonin and Makarova 2013).

Apart from the type I-E Cas7 structure described above, two other structures of Cas7 enzymes from type I-A (Csa2) and I-D (Csc2) were solved (Lintner *et al.*^2011^; Hrle *et al.*^2014^). Additionally, the Csm3 protein of type III-A as well as the Cmr4 protein of type III-B could be functionally and structurally linked to members of the Cas7 family (Hrle *et al.*^2013^; Ramia *et al.*2014a; Numata *et al.*^2015^). All these structures reveal a similar domain arrangement, consisting of the modified RRM (palm domain), the helical finger domain and the thumb domain. The exact structure of the I-A Cas7 and III-A Csm3 thumb domain is not known as the disordered region suggests the presence of a flexible loop. Mutations within the thumb domain decreased the crRNA affinity in both proteins (Lintner *et al.*^2011^; Hrle *et al.*^2013^). In contrast, the thumb of I-D Cas7 showed no direct influence on crRNA interaction, while mutations in the conserved palm domain interfered with RNA binding (Hrle *et al.*^2014^). All Cas7 members are suggested to provide the crRNP platform by oligomerizing on the crRNA, which protects the crRNA from degradation and enables hybridization with the target nucleic acids (van der Oost *et al.*^2014^). Furthermore, the Cas7 members of type III systems are endoribonucleases that catalyze the target cleavage (Ramia *et al.*2014a; Staals *et al.*^2014^; Tamulaitis *et al.*^2014^). Other members of the Cas7 family are found in type I systems (I-B, I-C and I-F) as well as type III systems (Csm5, Cmr6 and Cmr1) that share structural and sequence similarities with Cas7 (Makarova *et al.*2011a; Benda *et al.*^2014^).

The superimposition of available Cas5 structures of I-E (Cas5e), I-C (Cas5d), III-A (Csm4) and III-B (Cmr3) highlights a conserved core consisting of a modified RRM and a thumb domain (Nam *et al.*2012a; Koo *et al.*^2013^; Shao *et al.*^2013^; Jackson *et al.*2014a; Numata *et al.*^2015^). In all cases, a conserved binding pocket was identified that is most likely involved in the recognition of the specific crRNA 5^′^ handle (Jackson *et al.*2014a). Remarkably, some of the Cas proteins in the CRISPR-Cas systems can functionally be replaced. One example is the substitution of Cas5d for Cas6 in type I-C crRNP complexes. The evolution of this subunit is reflected in the modulation of the Cas5d structure as the Cas5 core is extended at its C-terminus with a repeat-specific endonuclease active site (Nam *et al.*2012a). A similar phenomenon is observed for the structure of Cmr3, which contains a second RRM domain at the C-terminus that is likely involved in the crRNP assembly process (Shao *et al.*^2013^). Additional candidates for Cas5 family members are found in the other type I systems (I-A, I-B, I-D and I-F). These proteins share a similar structural core, are proposed to recognize the crRNA 5^′^ handle and closely interact with the large subunit (Cas8, Cse1, Csy1 or Cas10) in the respective systems (Makarova *et al.*2011a; Shao *et al.*^2013^; Jackson *et al.*2014a).

Members of the Cas6 family show a two RRM domain arrangement and are experimentally characterized as crRNA-processing endonucleases in all type I and III systems, even though they show structural variations and a high-sequence divergence (Reeks, Naismith and White 2013; Niewoehner, Jinek and Doudna 2014). In the Cascade context, the I-E Cas6 is delivering the mature crRNA and tightly caps the 3^′^ handle (Sashital, Jinek and Doudna 2011; Jackson *et al.*2014a). This protection results in crRNAs with a complete 3^′^ handle, which was also shown for type I-F (Haurwitz *et al.*^2010^). In contrast, crRNAs from other type I (I-A, I-B, I-D) systems and all type III systems were shown to harbor trimmed 3^′^ ends. Consequently, the corresponding Cas6 enzymes are not likely to be permanent subunits of their crRNP complexes, but are rather stand-alone ribonucleases (Hatoum-Aslan, Maniv and Marraffini 2011; Richter *et al.*2012b; Plagens *et al.*^2014^). The varying affinities of Cas6 to mature crRNA products might be a consequence of the presence or absence of stable stem-loop structures in the crRNA 3^′^ handle or structural variations of Cas6 (Kunin, Sorek and Hugenholtz 2007; Niewoehner, Jinek and Doudna 2014). The diversification of Cas6 enzymes correlates with repeat sequence variation. However, the reasons for the evolution of different mechanisms of Cas6-mediated repeat cleavage and crRNA delivery to the crRNP complexes are not yet understood.

### Diversification of Cas proteins responsible for interference in type I and III CRISPR-Cas systems

The large and small subunits of different type I and type III crRNP complexes show limited sequence conservation and little structural similarities (Makarova *et al.*2011a; Makarova, Wolf and Koonin 2013). The large subunits comprise members of the Cas8 (I-A, I-B, I-C) and Cas10 (I-D, III-A, III-B) family as well as Cse1 and Csy1. In general, the classification of the large subunits is mainly based on their size, their function in PAM recognition and R-loop formation and/or their position in the crRNP complex (van der Oost *et al.*^2014^). Comparison of the two available structures of Cse1 of the I-E system and Cas10 of subtype III-B did not reveal structural similarities. The overall Cse1 structure has a unique globular fold with a coordinated zinc-ion and a C-terminal four-helix bundle (Mulepati, Orr and Bailey 2012; Jackson *et al.*2014a). In contrast, Cas10 has a four-domain protein core with two adenylyl cyclase-like domains and a permuted HD domain (Cocozaki *et al.*^2012^). Computational analyses predicted a common arrangement of core cyclase/polymerase-like domains including finger, palm and thumb domains for all large subunits (Makarova *et al.*2011a). Additionally, numerous members of the Cas8, Cas10d, Cas10 and Cse1 groups were suggested to contain a little conserved RRM-fold inside the palm domain (Makarova *et al.*2011b). However, Cse1 lacks a palm-like RRM and shared no common architectural layout with Cas10. One reason for the missing structural similarities of the diverse large subunits might be extensive structural rearrangements of the proteins (Koonin and Makarova 2013).

Similarly, only very limited structural conservation was observed for the small subunits of different crRNP complexes. Structures of the small subunits have been solved for type I-E (Cse2), I-A (Csa5) and subtype III-B systems (Cmr5) (Agari *et al.*^2008^; Sakamoto *et al.*^2009^; Park *et al.*^2013^; Reeks *et al.*2013a). In general, all these proteins are characterized by the presence of several α-helices, but show only very limited conservation between the N-terminal domains of Cse1 and Cmr5 and the C-terminal domains of Cse1 and Csa5 (Reeks *et al.*2013a). To further complicate this issue, it has been proposed that the small subunit is fused to the large subunits in several subtypes (I-B, I-C, I-D, I-F). Even within the same subtype, small subunits can show only very limited sequence similarity as observed for Csa5 of type I-A (Makarova *et al.*2011a; Daume, Plagens and Randau 2014).

Several reasons can be discussed for this phenomenon of Cas protein diversification. The proteins that interact with the crRNA, namely members of the Cas5, Cas6 and Cas7 families, likely show structural similarities, as the crRNA component is similar in all different subtypes (Makarova *et al.*2011a). In all type I and III systems, the 8-nt 5^′^ handle and a similar crRNA length is observed, which indicates an evolutionary conservation of the primary repeats within the host CRISPR loci (Kunin, Sorek and Hugenholtz 2007). Minor structural differences might be a consequence of the adaptation to variable prokaryotic growth parameters in the environment. In contrast, the large and small subunits interact with the respective targets in the surveillance complex. One obvious reason for different Cas protein target recognition mechanisms is the presence of either dsDNA or ssRNA as target molecules (van der Oost *et al.*^2014^). To effectively scan a compatible target, the respective proteins have to evolve specialized binding patches for either R-loop formation or the hybridization of RNA:RNA duplexes (Benda *et al.*^2014^; Mulepati, Heroux and Bailey 2014). Another aspect to consider is the high selective pressure for the continuous effectiveness of the crRNP surveillance. Different studies report evidence for the arms race of viruses and their prokaryotic host (Levin *et al.*^2013^; Seed *et al.*^2013^; Fineran *et al.*^2014^). This means that diverse crRNP complexes have likely evolved in reaction to viral measures to avoid crRNA-mediated targeting. One way to escape targeting is the introduction of mutation in the region targeted by the crRNA guide, which is most efficient for the seed sequence region. Alternatively, the introduction of variations in the PAM can also inhibit base pairing with the target (Deveau *et al.*^2008^; Westra *et al.*^2013^). Interestingly, recent experiments show that Cascade does not only bind targets tightly, but also establishes ‘low fidelity’ interactions with mutated targets independent of PAM or seed sequences. This process is proposed to allow the acquisition of new spacers (Blosser *et al.*^2015^). Another striking example of this arms race are phage-encoded anti-CRISPR genes that enable viruses to evade the CRISPR-Cas systems (Bondy-Denomy *et al.*^2013^; Pawluk *et al.*^2014^). Thus, the large and small subunits, as a central point in the surveillance machinery, have to adapt to these target variations and viral evasion strategies, possibly providing an explanation for their high degree of diversification. Finally, different mechanisms of target degradation are identified in the type I and III systems. In type I-E systems, Cas3 docks to the large subunit Cse1 of Cascade after R-loop formation, while the type I-A Cas3 is proposed to be an integral part of the complex (Hochstrasser *et al.*^2014^; Plagens *et al.*^2014^). The interaction of Cas3 to Cascade is mediated by its CTD. As a possible consequence of structural variations of the large subunits, Cas3 sequences show remarkable diversification of the CTD, despite harboring a highly conserved common core (Huo *et al.*^2014^; Jackson *et al.*2014b). In type III systems, the cleavage of the target is executed by the Cas7-like protein Cmr4 in the Cmr complex or Csm3 in the Csm complex (Rouillon *et al.*^2013^; Benda *et al.*^2014^; Staals *et al.*^2014^; Tamulaitis *et al.*^2014^). These observations highlight examples for striking differences among the crRNP complexes. Future studies on both CRISPR-Cas systems and their natural virus and plasmid targets should provide us with a better understanding of the coevolution and diversification of these antagonists.
